# Covalently attached intercalators restore duplex stability and splice-switching activity to triazole-modified oligonucleotides†

**DOI:** 10.1039/d2cb00100d

**Published:** 2022-05-16

**Authors:** Anna Dysko, Ysobel R. Baker, Graham McClorey, Matthew J. A. Wood, Sabine Fenner, Glynn Williams, Afaf El-Sagheer, Tom Brown

**Affiliations:** Department of Chemistry, Chemistry Research Laboratory, University of Oxford 12 Mansfield Road Oxford, OX1 3TA UK tom.brown@chem.ox.ac.uk; Department of Physiology, Anatomy and Genetics, University of Oxford Oxford UK; GSK Medicines Research Centre, Gunnels Wood Road, Stevenage Hertfordshire SG1 2NY UK; Chemistry Branch Department of Science and Mathematics, Faculty of Petroleum and Mining Engineering, Suez University Suez 43721 Egypt

## Abstract

Oligonucleotides are rapidly emerging as powerful therapeutics for hard to treat diseases. Short single-stranded oligonucleotides can base pair with target RNA and alter gene expression, providing an attractive therapeutic approach at the genetic level. Whilst conceptually appealing, oligonucleotides require chemical modification for clinical use. One emerging approach is to substitute the phosphodiester backbone with other chemical linkages such as triazole. The triazole linkage is inherently resistant to enzymatic degradation, providing stability *in vivo*, and is uncharged, potentially improving cell-penetration and *in vivo* distribution. Triazole linkages, however, are known to reduce RNA target binding affinity. Here we show that by attaching pyrene or anthraquinone to the ribose sugar on the 5′-side of the triazole, it is possible to recover duplex stability and restore the splice switching ability of triazole-containing oligonucleotides.

## Abstract 2

Oligonucleotides can bind to mRNA and alter gene expression, but require backbone modifications for clinical use. We show that attaching pyrene or anthraquinone to the ribose sugar next to an artificial triazole backbone restores duplex stability and splice switching ability in cells.

## Graphical abstract

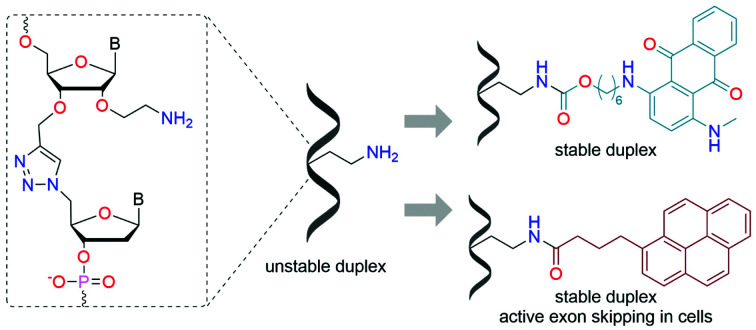

## Introduction

Antisense oligonucleotides (ASOs) are rapidly emerging as powerful agents for treating genetic diseases.^1–3^ These short single stranded analogues of DNA (typically 15–25 bases in length) can bind to their complementary RNA targets and alter gene expression by a variety of mechanisms.^4^ ASOs can inhibit translation of specific proteins by recruiting ribonuclease H1 (RNase H) to degrade the target messenger RNA (mRNA),^5^ or by binding to mRNA translational start sites, thereby blocking the binding of ribosomes.^6^ ASOs can also be designed to bind precursor mRNA (pre-mRNA) and modulate RNA splicing.^7–9^ This can introduce an out-of-frame deletion, triggering nonsense mediated decay of the transcript. Alternatively, it can correct aberrant splicing caused by splice point mutations, or truncate the mRNA by removing exons that contain premature stop codons or out-of-frame deletion mutations.

Without chemical modification, oligonucleotides (ONs) are rapidly degraded by nucleases in biological environments rendering them ineffective as therapeutics. For this reason, chemical modifications have been developed to provide protection against nucleases and enhance pharmacokinetic properties.^10^ Commonly used chemical modifications for ASOs include replacing phosphodiester (PO) linkages in the backbone with phosphorothioate groups (PS)^11,12^ to improve duplex stability against enzymatic degradation and cellular uptake, and ribose modifications to improve target affinity and lipophilicity. These include 2′-F, 2′-OMe, 2′-*O*-(2-methoxyethyl) and locked nucleic acids (LNAs).^11,13–15^ These modifications have transformed the field, and the FDA has now approved several ASOs for clinical use.^16^ However, the widespread therapeutic applications of ASOs are still hampered by potential toxicity and their inability to efficiently cross cellular membranes. Hence, there remains a need for new ASO chemistries to reduce dosage, toxicity, cost and improve clinical efficacy.

**Fig. 1:**
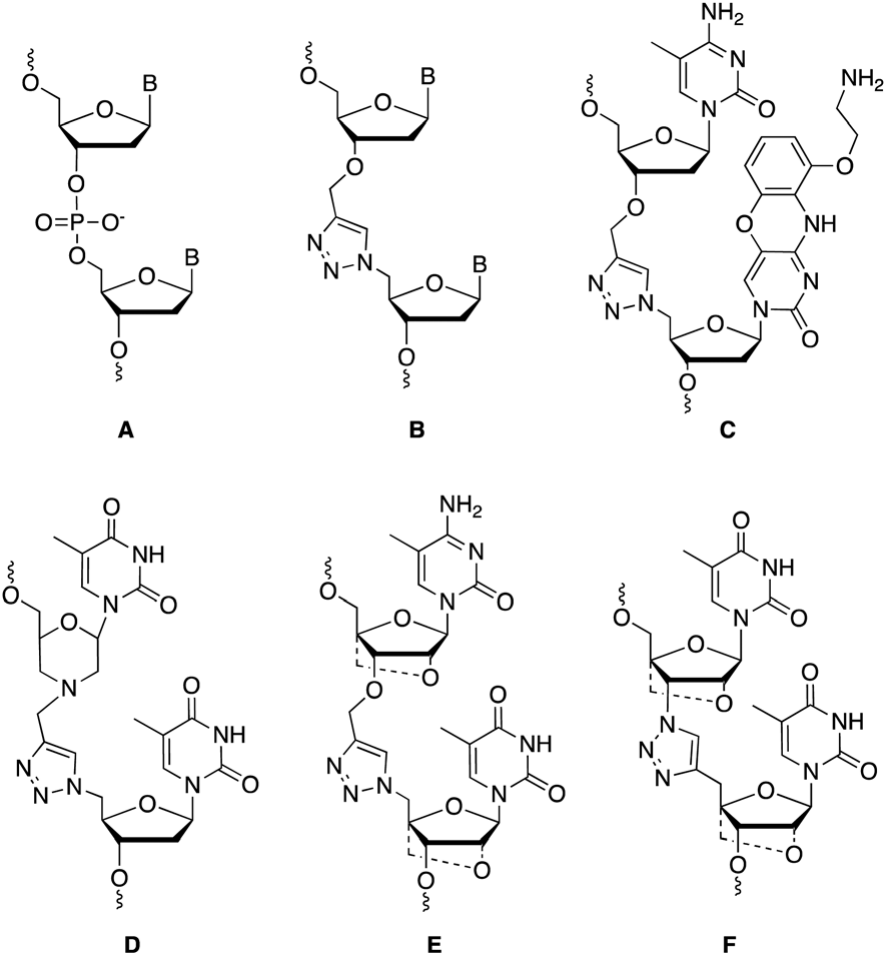
Comparison of the PO backbone (A) and the biocompatible triazole linkage (B). Current approaches to stabilise the linkage include using a G clamp (C) and replacing the ribose with a morpholino (D) or an LNA sugar (E and F), dashed bonds are used to indicate different combinations of LNA on the 5′ or 3′ were investigated.

Charge-neutral backbone modifications offer an intriguing alternative for ASO development.^17–19^ Here, the PO backbone is partially or fully replaced with linkages that have no charge (each phosphodiester linkage carries an anionic charge, Fig. 1A). These linkages can confer nuclease resistance (*in vivo* stability) and alter the overall anionic nature and lipophilicity of an ASO, potentially aiding cellular uptake and modulating protein interactions that influence *in vivo* distribution. The triazole linkage (Fig. 1B) has several characteristics that are desirable when developing new ASO candidates. It is biocompatible and completely stable to endogenous nucleases, it is simple to synthesise and is charge neutral.^20,21^ Despite these advantages, the triazole linkage reduces the affinity of an ON for target RNA, and a single triazole incorporation can significantly reduce the stability of ON : RNA duplexes.^21–23^ Several different approaches to overcome this have been reported including the use of an aminoethoxyphenoxazine nucleobase analogue (G-clamp) which increases duplex stability by a combination of increased base stacking and an extra hydrogen bond with guanine (Fig. 1C),^23^ and substitution of the flanking ribose sugars with a morpholino analogue (Fig. 1D),^24^ or an LNA derivative (Fig. 1E and F).^21,25,26^ Whilst several triazole linkage designs exist,^22,27–29^ this study focuses on the structure shown in Fig. 1B due to its ease of synthesis. In the current study, by incorporating various chemical moieties adjacent to the triazole linkage that are known to stabilise ON duplexes, we have significantly improved the RNA binding affinity of ONs.

## Results and discussion

### Synthesis of triazole modified ONs

**Fig. 2:**
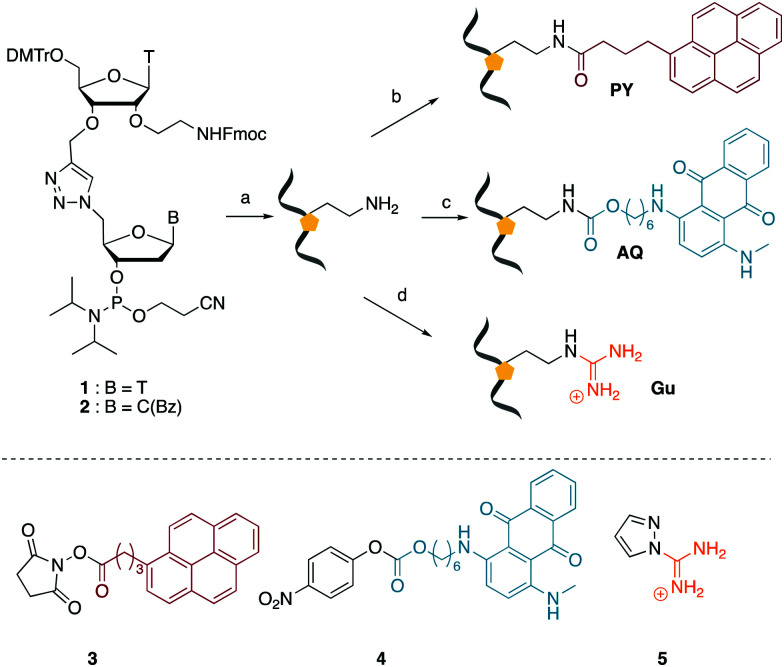
(Top) Overview of ON synthesis strategy *via* amino-modified ONs. (Bottom) reagents used to functionalise the ONs. *Reagents and conditions*: (a) solid phase ON synthesis, (b) *N*-hydroxysuccinimide (NHS) labelling with 3, (c) nitrophenyl active ester (oNp) labelling with 4, (d) guanidine addition with 5. DMT = 4,4′-dimethoxytrityl.

We envisaged the development of universal phosphoramidite building blocks that would enable multiple incorporations of the triazole and allow the post-synthetic attachment of various chemical functionalities to the 2′-position of the ribose on the 5′-side of the triazole. The phosphoramidites (Fig. 2) were designed to be compatible with standard solid-phase ON synthesis and contain an amino group that can be functionalised after ON synthesis. A key advantage to this approach is that one phosphoramidite monomer can be used to screen multiple modifications without requiring multistep synthesis of a new phosphoramidite for each modification of interest. In this study, three different moieties were investigated: pyrene (PY) and anthraquinone (AQ) which are known to increase duplex stability by intercalation and/or minor grove binding,^30–37^ and guanidine (Gu) which stabilises duplexes by masking the charge repulsion between opposite nucleic acid strands, and potentially improves ON cellular uptake.^38–40^ Although aromatic groups such as pyrene or anthraquinone stabilise unmodified duplexes due to intercalation or groove binding, in this study, we specifically wanted to determine if they could increase duplex stability when placed directly next to an artificial backbone. We also wanted to determine if cationic guanidine stabilises duplexes when placed adjacent to an uncharged artificial backbone.

**Scheme 1:**
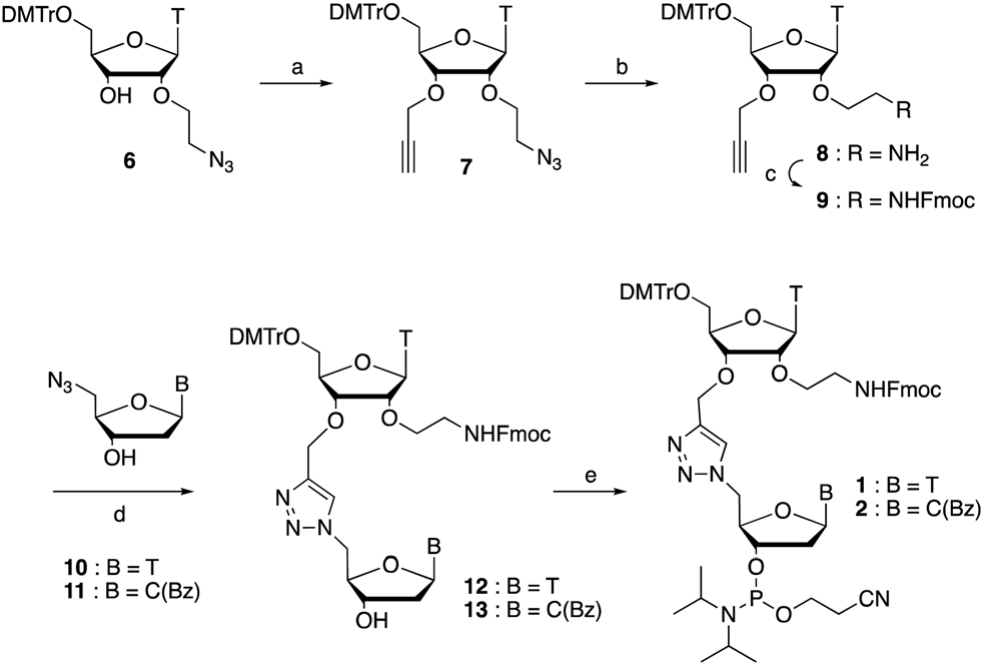
Synthesis of phosphoramidites 1 and 2. (a) NaH, propargyl bromide, THF, reflux, 14 h, 72% (b) Ph_3_P, H_2_O, THF, 45 °C, 2 h (c) Fmoc-OSu, CH_2_Cl_2_, pyridine, r.t., 2 h, 65% over 2 steps (d) sodium ascorbate, CuSO_4_, (tris-(benzyltriazolylmethyl)amine ligand (TBTA), DMF, H_2_O, r.t., 4 h, T = 86%, C(Bz) not fully purified. (e) 2-cyanoethyl-*N*,*N*-diisopropylchlorophosphoramidite, DIPEA, CH_2_Cl_2_, r.t., 2.5 h, T = 34%, C(Bz) = 55% over 2 steps.

The synthesis of phosphoramidites 1 and 2 started with propargylation of the 2′-hydroxy group in ribonucleoside 6^41^ (Scheme 1). Staudinger reduction of the azide in 7 followed by protection of the amine in 8 using Fmoc-succinimide (Fmoc-OSu) gave nucleoside 9. This was then joined to 5′-azidothymidine 10^42^ or benzoyl protected 5′-azidodeoxycytidine 11^43^ using the Cu^I^-catalysed alkyne-azide cycloaddition (CuAAC) reaction^44^ to give the T–T dinucleoside 12 and the T–C dinucleoside 13 respectively. These were then phosphitylated to give the phosphoramidite building blocks 1 and 2.

We then evaluated the labelling strategy shown in Fig. 2. ON1 (Table 1) was synthesised using phosphoramidite 1 and further functionalised. Commercially available 1-pyrenebutyric acid *N*-hydroxysuccinimide (NHS) ester 3 was used to attach pyrene with a butyryl linker to yield ON2. ON1 was converted to ON3 using the active carbonate derivative of AQ 4 which was prepared from the previously reported alcohol^45,46^ using 4-nitrophenyl chloroformate. ON1 was guanadinylated using 1*H*-pyrazole-1-carboxamidine hydrochloride to give ON4. Details for the labelling conditions and synthesis of 4 are given in the supporting information. A second set of ONs with a phosphodiester instead of a triazole linkage (ON5-8, Table 1) was prepared for use as controls in the biophysical studies. ON5 was synthesised using a 2′-aminoethoxy thymidine (AE–T) phosphoramidite^41^ and labelled with NHS ester 3, active carbonate 4 and carboxamidine 5, as described above, to give ONs 6–8 respectively.

**Table 1:** Sequences of ONs and their melting temperatures (*T*_m_) when hybridised with complementary DNA and RNA

ON	Sequence 5′ to 3′	DNA target *T*_m_ (Δ*T*_m_) °C	RNA target *T*_m_ (Δ*T*_m_) °C
1	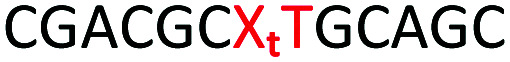	53.0 (−8.9)	54.6 (−7.4)
2	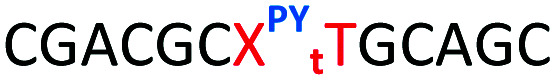	66.0 (+4.1)	61.3 (−0.7)
3	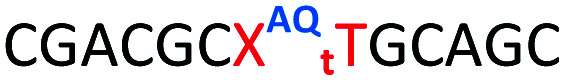	66.1 (+4.2)	64.6 (+2.6)
4	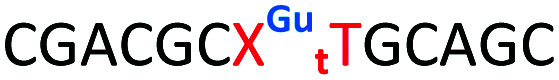	53.4 (−8.5)	54.2 (−7.8)
5	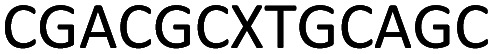	59.0 (−1.9)	60.5 (−1.5)
6	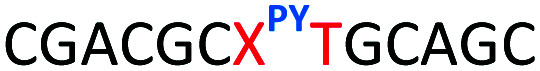	68.4 (+6.5)	64.5 (+2.6)
7	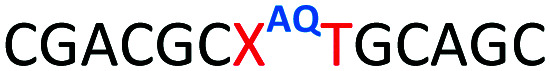	69.5 (+7.6)	69.0 (+7.0)
8	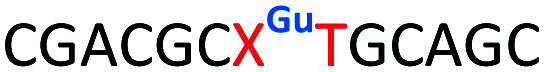	61.5 (−0.4)	62.8 (+0.8)
9	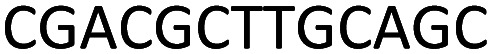	61.9	62

### Biophysical evaluation

#### Single addition

**Fig. 3:**
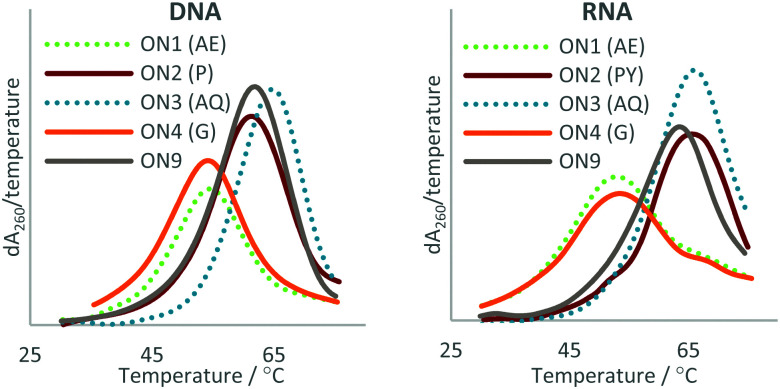
UV melting studies of the triazole-modified ONs against DNA (Left) and RNA (Right). The first derivatives of the melting curves are shown. AE = 2′-aminoethoxy, P = pyrene, AQ = anthraquinone, G = guanidine. For sequences see Table 1.

UV melting was used to determine the melting temperatures (*T*_m_s) (duplex stabilities) of ONs 1–9 with complementary DNA and RNA targets. The *T*_m_ values are given in Table 1 and melting derivatives of the modified duplexes are shown in Fig. 3.

The data show that this approach can be used to overcome the loss in duplex stability associated with backbone modifications^47^ such as the charge-neutral triazole. In agreement with previous reports, the triazole is destabilising compared with the phosphodiester linkage,^21–23^ a Δ*T*_m_ of −8.9 °C was observed in duplexes with DNA and a Δ*T*_m_ of −7.4 °C in duplexes with RNA (compare ON1 with ON9). This indicates that the 2′-aminoethoxy (AE) modification alone does not significantly improve the duplex stability of triazole modified ONs. Attachment of PY and AQ resulted in DNA duplexes that were 4.1 and 4.2 °C more stable than the unmodified DNA : DNA duplex (compare ON2 : DNA and ON3 : DNA with ON9 : DNA). PY and AQ also regained the loss in thermal stability of duplexes with a complementary RNA target, albeit to a lesser degree. The addition of PY to the triazole resulted in a duplex with RNA that was 0.7 °C less stable than the unmodified duplex (compare ON9 : RNA with ON2 : RNA) but 6.7 °C more stable than the triazole modified ON1 (compare ON2 : RNA with ON1 : RNA). AQ improved the stability of the triazole modified DNA : RNA duplex to a greater degree than PY, with an increase in *T*_m_ of 2.6 °C compared to unmodified DNA (compare ON3 : RNA with ON9 : RNA) and an increase of 10 °C compared to the triazole modified DNA (compare ON3 : RNA with ON1 : RNA). It is worth noting that the Gu modification had little effect on the stability of duplexes with and without a triazole linkage, and was therefore not considered further. Guanidine is cationic at neutral pH, and stabilises unmodified duplexes by masking charge repulsion between opposite nucleic acid strands. However, in the modified duplexes guanidine is close to an uncharged triazole linkage. It is likely that stabilisation does not occur because there is no ‘anion–anion’ charge repulsion to neutralise at triazole site.

These findings suggest that it is not the addition of the PY and AQ modifications alone, but synergy between the triazole and the PY/AQ modifications that give rise to the observed stabilisation. For example, looking at destabilisation resulting from triazole (ON1 : DNA Δ*T*_m_ = −8.9 °C) and the stabilising effect of the PY without the triazole (ON6 : DNA Δ*T*_m_ = +6.5 °C) it might be expected that a combination of the two modifications would produce a duplex with slightly lower stability than the unmodified control duplex; however, a significant increase in stability is observed (+4.1 °C). The triazole only destabilises a pyrene modified ON by −2.4 °C (compare ON6 : DNA with ON2 : DNA) which is much lower than the −8.9 °C difference between triazole-modified ON1 : DNA and the control ON9 : DNA. Similar results were observed for the AQ modification against DNA. This effect was also observed for the modified ONs with RNA but to a lesser degree.

The duplex stabilisation conferred by the triazole-pyrene or triazole-anthraquinone oligonucleotides against DNA (Table 1) is slightly greater than against RNA. However, in these examples the majority of the oligonucleotide backbone is deoxyribose-phosphodiester (*i.e.*, natural DNA), which is relevant to a number of diagnostic applications including real-time PCR. However, the application described below involves cell studies, where the target is RNA, and for which stability to nucleases is essential. For this reason, we used a 2′-OMe RNA-phosphorothioate backbone which is known to favour binding to RNA targets over DNA targets, so *in vivo* selectivity of the triazole-modified oligonucleotides for RNA over DNA should not be an issue.

**Fig. 4:**
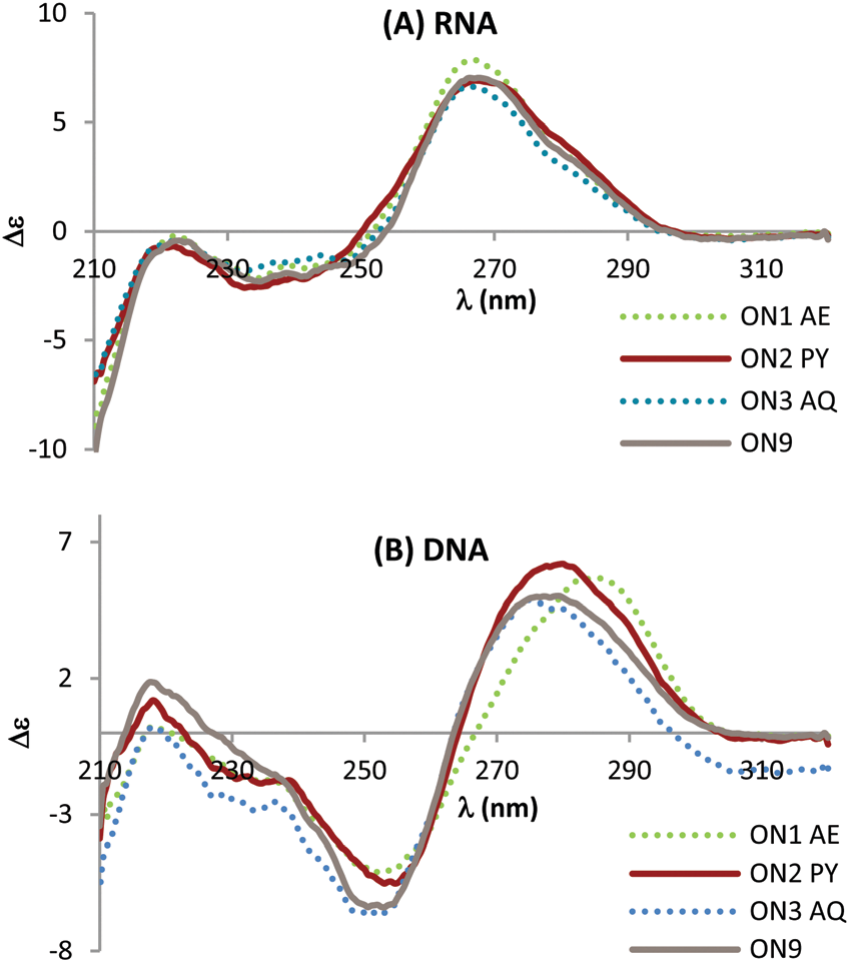
CD spectra of the modified DNA : RNA duplexes (top) and modified DNA : DNA duplexes (bottom). For sequences see Table 1. Melting temperatures were determined at 4 μM duplex concentration in 10 mM NaH_2_PO_4_/Na_2_HPO_4_ buffer with 200 mM NaCl at pH 7.2. Sequence of DNA target = GCTGCAAGCGTCG; sequence of RNA target = GCUGCAAGCGUCG.

Circular dichroism (CD) studies show that triazole combined with PY or AQ has greater effect on the structure of DNA : DNA duplexes than the corresponding DNA : RNA duplexes (Fig. 4) suggesting that the mode of interaction in the duplexes with a DNA target could be different to that for duplexes with an RNA target. This could explain the additional stability observed when duplexed with DNA compared to RNA. When hybridised with complementary RNA, ON1–ON3 show similar CD spectra, and they overlap with the unmodified ON9 : RNA spectra almost perfectly (Fig. 4A). These constructs display characteristic A-type signals with strong maxima at 270 nm and minima at 240 nm. In contrast, whilst the CD spectra for ON1-3 retain B-type features (maxima at 280 nm and minima at 250 nm) variations in the shift and peak intensity were observed compared to the unmodified duplex. These observations suggest that the triazole perturbs the B form structure and that the AQ and PY modifications restore the B form (as observed by the slight blue shift in the peak at 280 nm). Further structural studies are required to determine whether this is a result of intercalation or groove binding.

#### Multiple additions and different backbones

A combination of 2′-OMe and phosphorothioate (PS) is often used to improve the properties of ASOs *in vivo.* Hence, we evaluated the effect of single and multiple triazole linkages on RNA binding affinity in a model ASO sequence with a 2′-OMe/PS backbone.^48^ The ONs (Table 2) with 1, 3, or 4 incorporations of the triazole linkage were synthesised using phosphoramidite dimers 1 and 2, which were fully compatible with the sulfurisation step used during solid-phase ON synthesis. The ONs were then labelled with PY-NHS active ester 3 as described earlier to give ONs 13–15 or with AQ-*o*-nitrophenyl active carbonate 4 to give ONs 16–18.

**Table 2:** Thermal melting data and sequences of the PS/2′-OMe ONs used in assay 1. Melting temperatures against complementary RNA

ON	ON sequence (5′ to 3′)	*T*_m_ °C
10	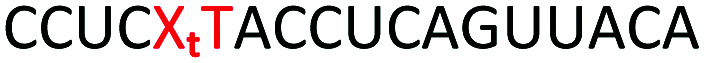	65.9
11	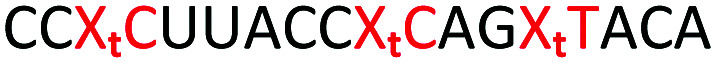	55.5
12	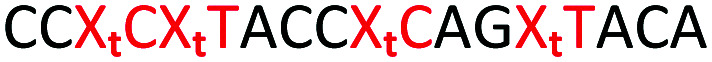	49.4
13	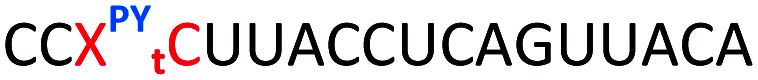	71.1
14	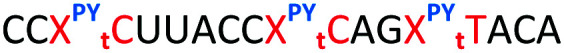	60.5
15	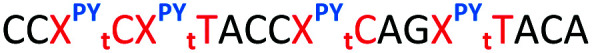	56.7
16	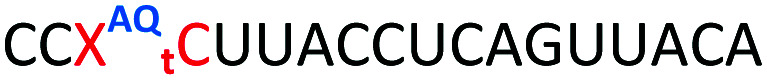	74.1
17	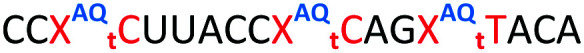	51.1
18	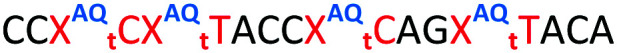	45.1
19	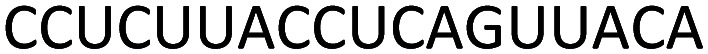	70.2

The *T*_m_s of the ONs with a complementary RNA strand were determined by UV-melting (Table 2). Incorporation of a single triazole linkage (ON10) decreased the thermal stability of the duplex by 4.3 °C, triple addition (ON11) reduced the *T*_m_ by 14.7 °C and quadruple addition (ON12) dropped the *T*_m_ by 20.8 °C. These results might suggest that the triazole is better tolerated in an A-form PS/2′-OMe backbone than a B-form DNA backbone (compare ON1 : RNA Δ*T*_m_ = −7.4 °C with ON10 : RNA Δ*T*_m_ = −4.3); however, as these data were recorded in different sequences caution must be applied when drawing comparisons. As before, addition of PY and AQ to the triazole modified ONs restored some of the lost stability. ON13 with a single triazole linkage and PY showed a *T*_m_ increase of 5.2 °C compared to the triazole alone in ON10 and exceeded the *T*_m_ of the PS/2′-OMe control ON19 without a triazole by 0.9 °C. Similarly, addition of AQ to ON10 increased the stability by 8.2 °C (Compare ON10 with ON16), which is 3.9 °C higher than the unmodified control ON19.

The stabilising effect of AQ and PY with triple and quadruple additions of the triazole linkage was not as great as anticipated. Adding PY to ON11 with three triazole linkages only increased the *T*_m_ by 5.0 °C (ON14), and the resulting duplex was 9.7 °C less stable than the control (ON19). ON15 with four PY functionalised triazole linkages was only 7.3 °C more stable than ON12 without PY, and 13.5 °C less stable than the control. More surprisingly, addition of three and four anthraquinone moieties next to triazole linkages (ON17 *T*_m_ = 51.1 °C and ON18 *T*_m_ = 45.1 °C) caused a decrease in duplex stability compared to the triazole containing ON11 (*T*_m_ = 55.5 °C) and ON12 (*T*_m_ = 49.4 °C).

### Biological evaluation of the pyrene triazole combination

Having confirmed that pyrene is more capable than anthraquinone of compensating for the loss of duplex stability incurred with multiple triazoles in PS/2′-OMe ASOs, we evaluated efficacy of the modified ASOs in a commonly used splice-switching reporter cell system.^48^ This model system is based on a mutation that causes the blood disorder β-thalassemia. The HeLa pLuc705 cells used had been stably transfected with a plasmid that encodes for luciferase interrupted by a mutated human β-globin intron 2 (IVS2-705). This single point mutation causes aberrant splicing, preventing translation of active luciferase. Masking the aberrant splice site with an ASO redirects the splicing machinery and restores luciferase production. ONs used in the *T*_m_ studies (Table 2) were designed to be complementary to the splice site, and ON19 is the standard control sequence used with this assay.

**Fig. 5:**
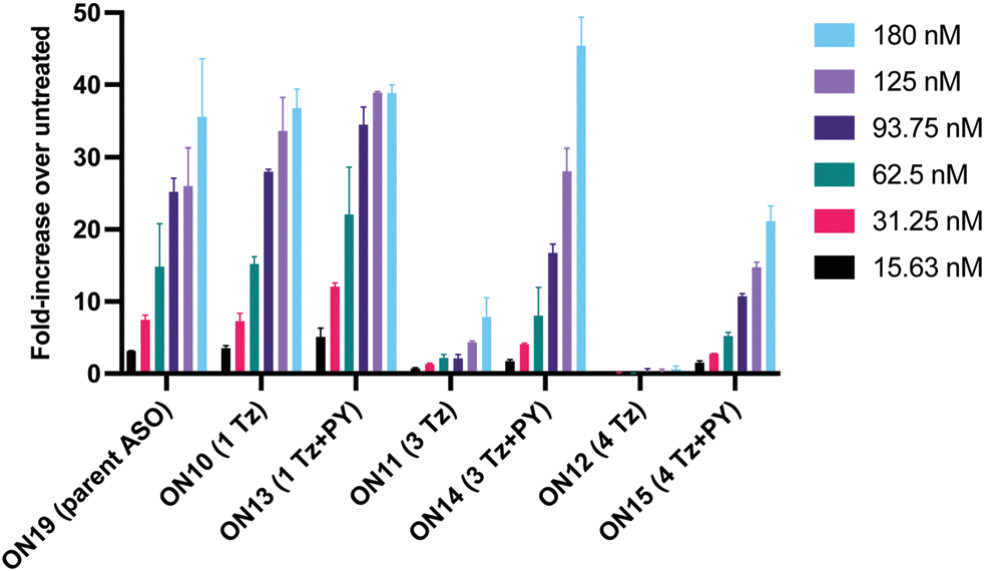
Splice-switching activity of transfected antisense oligonucleotides in the stably transfected HeLa Luc/705 cell line (assay 1) using Lipofectamine 2000. Cells were treated for 24 h. Experiments were replicated with data showing standard deviation of the mean (SD).

The splice switching assay confirmed that replacement of the PS backbone with more than one triazole is detrimental to activity and showed that the addition of pyrene to the triazole modified ASO re-establishes splice switching. The addition of PY also improved the response of the ASO with a single triazole relative to the parent ASO (Fig. 5). Here, the cells were treated with ON10-15 complexed with Lipofectamine 2000 in the concentration range 7.8 nM to 180 nM. At the higher concentration a maximal effect is observed for ON10, ON13, ON15 and ON19 (around 40-fold increase in activity). Higher doses of oligonucleotide-lipofectamine complexes caused significant cell toxicity. A clear dose response was observed for all oligonucleotides except for ON12 with four triazoles and no PY, which was inactive. These dose responses indicate that the most to least active oligonucleotides are ON13 (*T*_m_ = 71.1 °C) > ON10 (*T*_m_ = 65.9 °C) > ON19 (*T*_m_ = 70.2 °C) > ON14 (*T*_m_ = 60.5 °C) > ON15 (*T*_m_ = 56.7 °C) > ON11 (*T*_m_ = 55.5 °C) > ON12 (*T*_m_ = 49.4 °C) suggesting that duplex stability correlates with splice switching efficacy. It is clear that the addition of pyrene to triazole modified ONs improves their splice switching activity; a higher response is observed when comparing ON13 with ON10 at the lower concentrations, and ON14 and ON15 with PY modifications are significantly more active than their triazole-only equivalents ON11 and ON12.

**Fig. 6:**
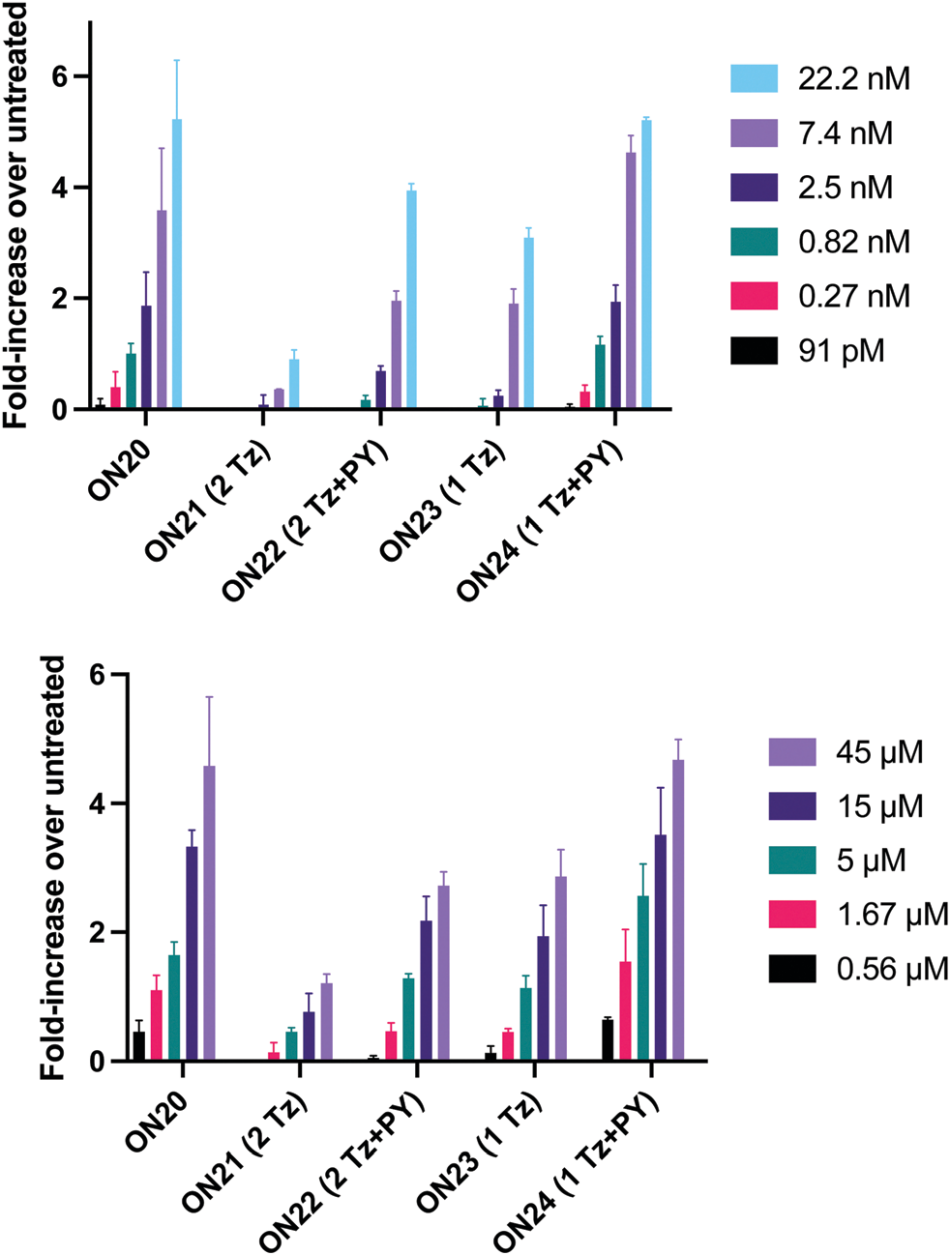
Splice-switching activity of triazole-modified ASOs in MCF7 cells transduced with a pHTBV1 plasmid encoding luciferase interrupted with IVS2-695 (assay 2). (Top) Cells were treated for 24 h with lipofectamine : ASO complexes. (Bottom) Gymnotic delivery experiments. Cells were treated with the ASO for 24 without a transfection agent. Experiments were performed in triplicate with data showing standard deviation (SD).

In order to substantiate these findings, a second luciferase exon skipping assay was performed, targeting the C to T nucleotide mutation at position 654 in human β-globin intron 2, a mutation that also causes β-thalassemia. In this assay, MCF7 cells are transduced with a pHTBV1 plasmid encoding luciferase, interrupted with IVS2-695, using BacMam one day prior to treatment with the ONs. The sequences of the ONs used and their duplex melting temperatures are shown in Table 3. As before, PY restored the duplex stability lost from the triazole incorporation, although some sequence dependence was observed. Lipofection with ON20-24 in a concentration range of 91 pM to 22.2 nM resulted in a dose dependent response for all ONs tested (Fig. 6, top graph) and toxicity was observed at higher concentrations. Again, the data show that the triazole alone reduces the efficacy of the ASO and that adding a PY restores the activity of the ASO. As before, a correlation between the *T*_m_ and activity was observed, with the exception of ON24 (*T*_m_ = 70.7 °C) appearing slightly more active that ON20 (*T*_m_ = 72.8 °C).

**Table 3:** Sequences and thermal melting data for the PS/2′-OMe ONs used in assay. Melting temperatures against complementary RNA

ON	ON sequence (5′ to 3′)	*T*_m_ °C
20	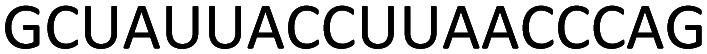	72.8
21	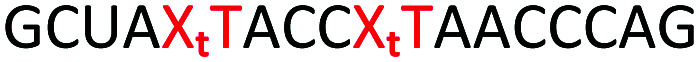	60.5
22	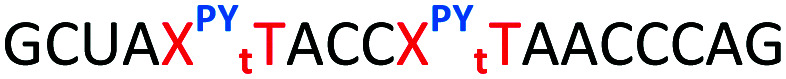	68.1
23	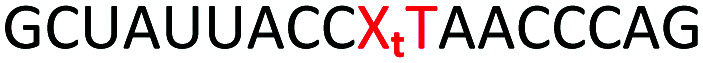	66.1
24	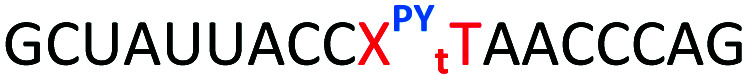	70.7

The effect of modifications on gymnotic (naked) delivery was also determined using this assay (Fig. 6 bottom graph). As with the lipofection experiments PY improved the activity of the triazole modified ONs. Double addition of the triazole without PY (ON21) also had a less detrimental effect on activity when compared to the parent ASO (ON20) than was observed with lipofection. This could be a result of increased lipophilicity aiding endosomal escape or cell uptake. Further investigations are required to clarify this.

## Conclusions

We have prepared two novel dinucleotide phosphoramidite monomers containing a triazole internucleoside linkage and a 2′-aminoethoxy modification on the 5′-side of the triazole. The building blocks were readily incorporated into oligonucleotides during solid phase oligonucleotide synthesis and provided a convenient point for post-synthetic functionalisation with electrophilic reagents. The effects on duplex stability of cationic and intercalating agents positioned adjacent to the triazole linkage were then compared. Having identified that addition of the AQ or PY modifications restores the stability lost by inclusion of the triazole, we evaluated these linkages in exon-skipping ASOs using two splice switching assays. As anticipated, the triazole alone resulted in lower activity; however, addition of PY to the ONs fully or partially restored the lost activity and, in some cases, the combination resulted in more potent ASO than the parent ASO with a standard 2′-OMe-phosphorothioate backbone. Taken together, these results indicate that the triazole linkage combined with PY or AQ could be a useful addition to the ASO chemical toolkit and is worth further exploration. Placing the modifications in the gap or the wings of antisense oligonucleotides, is also worth investigating to determine if they interfere with the activity of RNase-H. Use of the modifications in siRNA might also feasible. In general, future research, including optimisation of the linkage between the nucleoside and the PY or AQ, might produce oligonucleotides with increased potency.

The approach used here can also potentially be applied to other backbone modifications which may improve an ASO's pharmacokinetic properties but have previously taken a backseat due to duplex stability challenges. It also provides a route for the rapid screening of chemical attachments, beyond the three functionalities presented here, and could be used to evaluate a plethora of moieties for endosomal escape and/or enhanced duplex stability.

## Conflicts of interest

There are no conflicts to declare.

## Supplementary Material

CB-003-D2CB00100D-s001
