# Supplementary material for: Covalently attached intercalators restore duplex stability and splice-switching activity to triazole-modified oligonucleotides
Source: RSC Chem Biol. 2022 May 16;3(6):765–72. doi: 10.1039/d2cb00100d (PMC9175110; doi:10.1039/d2cb00100d)
Supplement: CB-003-D2CB00100D-s001 [file CB-003-D2CB00100D-s001.pdf]

Electronic Supplementary Material (ESI) for RSC Chemical Biology.
This journal is © The Royal Society of Chemistry 2022

Covalently attached intercalators restore duplex stability and splice-switching activity to triazole-modified oligonucleotides

Anna Dysko,[a] Ysobel R. Baker,[a] Graham McClorey,[c] Matthew J. A. Wood,[c] Sabine Fenner,[d] Glynn Williams,[d] Afaf El-Sagheer[a,b] and Tom Brown*[a]

Contents

Oligonucleotides used in this study 2

Small molecule synthesis 3

General 3

5′-*O*-(4,4′-Dimethoxytrityl)-3′-*O*-propargyl-2′-*O*-(2-azidoethyl) thymidine (7) 4

5′-*O*-(4,4′-Dimethoxytrityl)-3′-*O*-propargyl-2′-*O*-(2-aminoethyl) thymidine (8) 5

5′-*O*-(4,4′-Dimethoxytrityl)-3′-*O*-propargyl-2′-*O*-(2-(fluorenylmethoxyamido)ethyl)-thymidine (9) 5

2′-Aminoethoxy-*N*-Fmoc-5′-*O*-dimethoxytrityl 3′-thymidinyltriazoyl thymidine (12) 6

2′-Aminoethoxy-*N*-Fmoc-5′-*O*-dimethoxytrityl 3′-thymidinyltriazoyl benzoylcytidine (13) 8

2′-Aminoethoxy-*N*-Fmoc-5′-*O*-dimethoxytrityl-3′-thymidynyltriazoyl thymidine phosphoramidite(1) .9

2′-Aminoethoxy-*N*-Fmoc-5′-*O*-dimethoxytrityl-3′-thymidynyltriazoyl benzoylcytidine phosphoramidite (2) 10

1-(Methylamino)anthraquinone-4-[(6-*O*-(4-nitrophenyl)-hexyl)amino carbonate (4) 11

Oligonucleotide synthesis 12

General 12

Unmodified DNA strands 12

Unmodified RNA strands 13

Synthesis and purification of phosphodiester modified oligonucleotides using phosphoramidites 1 and 2. 14

Synthesis and purification of phosphorothioate modified oligonucleotides containing triazole linkages and 2′-aminoethoxy groups 14

Post synthetic functionalisation of modified oligonucleotides 15

Functionalisation with pyrene 3 or anthraquinone 4 15

Functionalisation with guanidine 15

Biophysical analysis 16

UV melting studies 16

Circular dichroism 16

Cell biology 16

General conditions 16

Comparing ASO activity using Hela pLuc705 cells. 16

Treatment of MCF7 cells with oligonucleotides 17

NMR spectra of compounds 19

Representative HPLC-mass spectra of oligonucleotides 27
References 32

Oligonucleotides used in this study

Table S1: List of phosphodiester/phosphorothioate oligonucleotides: X_tT = triazole dimer **1**, X_tC = triazole dimer **2**, X = 2′-aminoethoxy T, $_t$ = triazole linkage. X^{PY} = 2′-aminoethoxy T labelled with pyrene **3**, X^{AQ} = 2′-aminoethoxy T labelled with anthraquinone **4**, X^{Gu} = 2′-aminoethoxy T labelled with guanidine **5**

ON	Sequence 5′ to 3′	Calculated mass	Found mass
1	CGACGCX_tTGCAGC	3996	3995
2	CGACGCX^{PY}_tTGCAGC	4266	4266
3	CGACGCX^{AQ}_tTGCAGC	4374	4373
4	CGACGCX^{Gu}_tTGCAGC	4039	4038
5	CGACGCXTGCAGC	3995	3997
6	CGACGCX^{PY}TGCAGC	4265	4266
7	CGACGCX^{AQ}TGCAGC	4373	4374
8	CGACGCX^{Gu}TGCAGC	4038	4037
9	CGACGCTTGCAGC	3936	3936
10	CCUCX_tTACCUCAGUUACA	6109	6111
11	CCX_tCUUACCX_tCAGX_tTACA	6106	6107
12	CCX_tCX_tTACCX_tCAGX_tTACA	6118	6120
13	CCX^{PY}_tCUUACCUCAGUUACA	6381	6382
14	CCX^{PY}_tCUUACCX^{PY}_tCAGX^{PY}_tTACA	6916	6918
15	CCX^{PY}_tCX^{PY}_tTACCX^{PY}_tCAGX^{PY}_tTACA	7198	7200
16	CCX^{AQ}_tCUUACCUCAGUUACA	6487	6490
17	CCX^{AQ}_tCUUACCX^{AQ}_tCAGX^{AQ}_tTACA	7240	7242
18	CCX^{AQ}_tCX^{AQ}_tTACCX^{AQ}_tCAGX^{AQ}_tTACA	7632	7635
19	CCUCUUACCUCAGUUACA	6096	6098
20	GCUAUUACCUUAACCCAG	6160	6161
21	GCUAX_tTACCX_tTAACCCAG	6185	6185
22	GCUAX^{PY}_tTACCX^{PY}_tTAACCCAG	6725	6726
23	GCUAUUACCX_tTAACCCAG	6177	6173
24	GCUAUUACCX^{PY}_tTAACCCAG	6443	6443
T1	GCTGCAAGCGTCG DNA	3976	3976
T2	GCUGCAAGCGUCG RNA	4155	4156
T3	UGUAACUGAGGUAGAAGG RNA	5859	5859
T4	CUGGGUUAAGGUAAUAGC RNA	5796	5798

Small molecule synthesis

General

Chemicals/Procedures: All chemicals were purchased from Sigma-Aldrich, Alfa Aesar, Acros Organics or Fisher Scientific and used without further purification. When mentioned, solvents were degassed by bubbling argon through the solvent for 15 min. All air/moisture sensitive reactions were carried out under an inert argon atmosphere in oven-dried glassware. Unless otherwise stated, yields refer to analytically pure compounds.

Chromatography: Reactions were monitored by thin layer chromatography (TLC) using Merck Kieselgel 60 F_{254} aluminium-backed TLC plates. The compounds were visualised by UV irradiation at 254/365 nm and by staining in *p*-anisaldehyde or KMnO4. Column chromatography was carried out using Geduran Silica Gel 60 from Merck.

NMR Spectroscopy: ^{1}H, ^{13}C, ^{31}P NMR spectra were recorded using Bruker AVIII 400 MHz, Bruker AVII 500 (MHz or Bruker Neo 600 spectrometers using an internal deuterium lock at ambient probe temperatures. ^{1}H NMR chemical shifts (δ_H) are given to the nearest 0.01 ppm and are referenced relative to the appropriate residual solvent signal. Coupling constants (*J*) are given to the nearest 0.1 Hz. The following abbreviations are used to indicate the multiplicity of signals: s = singlet, d = doublet, t = triplet, q = quartet, m = multiplet, app = apparent, and br = broad. Data is reported as follows: chemical shift (integration, multiplicity, coupling constant(s)). ^{13}C NMR chemical shifts (δ_C) are quoted to the nearest 0.1 ppm and are reference relative to the deuterated solvent peak. ^{31}P NMR chemical shifts (δ_P) are given to the nearest 0.01 ppm. NMR assignments were supported by COSY, HMQC, and HMBC where necessary.

Mass Spectroscopy: Low-resolution mass spectra (LRMS) were measured on a Waters LCT premier mass spectrometer and Waters ZMD quadrupole mass spectrometer for +/- electrospray ionisation (ESI). High resolution mass spectra (HRMS) were recorded on a Thermo Scientific Exactive Mass Spectrometer equipped with a Waters Equity autosampler and pump by the University of Oxford

Chemistry Departmental Mass Spectrometry Service. Reported mass values are within ± 5 ppm mass units unless otherwise stated.

Infrared Spectroscopy: Infrared spectra were obtained using a Bruker 27 FT-IR spectrometer. Selected diagnostic absorption maxima (ν_{max}) are given in wavenumbers (cm^{-1}).

5′-*O*-(4,4′-Dimethoxytrityl)-3′-*O*-propargyl-2′-*O*-(2-azidoethyl) thymidine (7)

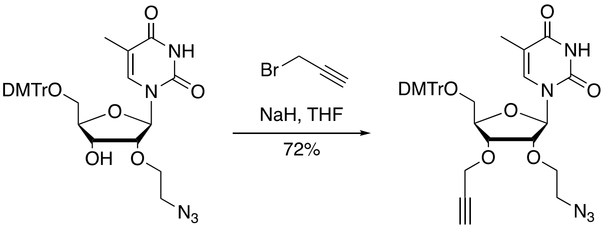

6 7

Alkyne **6**[1] (1.0 g, 1.6 mmol, 1.0 eq) was dissolved in anhydrous THF (7 mL) and cooled to 0 °C. Sodium hydride (60% dispersion in mineral oil) (0.16 g, 4.0 mmol, 2.5 eq) was added in four portions. After 1 h propargyl bromide (80% in toluene, 0.39 mL, 3.5 mmol, 2.2 eq) was added and the reaction was heated to reflux for 14 h. H_2O (0.5 mL) was added to quench the reaction and solvent was removed *in vacuo*. The residue was dissolved in CH_2Cl_2 (50 mL) and washed with water (2 x 50 mL) and brine (1 x 50 mL). The organic layer was dried over Na_2SO_4, filtered and the solvent removed *in vacuo*. The resulting crude material was purified by flash column chromatography on silica gel (1-3% MeOH in CH_2Cl_2 with constant 1% pyridine) giving compound **7** (0.765 g, 1.15 mmol) as a white foam in 72% yield.

δ_H (400 MHz, $CDCl_3$) 8.38 (1H, s), 7.72 (1H, d, *J* = 1.1), 7.38 (2H, d, *J* = 7.4), 7.34 – 7.11 (7H, m), 6.79 (4H, d, *J* = 8.7), 5.89 (1H, d, *J* = 2.2), 4.53 (1H, dd, *J* = 7.6, 5.0), 4.29 – 4.17 (3H, m), 4.14 (1H, dd, *J* = 5.0, 2.2), 4.04 (1H, dt, *J* = 10.1, 4.5), 3.82 (1H, dt, *J* = 10.1, 5.5), 3.74 (6H, s), 3.52 (1H, dd, *J* = 11.2, 2.0), 3.44-3.34 (3H, m), 2.38 (1H, t, *J* = 2.4), 1.29 (3H, d, *J* = 1.1).

δ_C (101 MHz, $CDCl_3$) 164.0, 158.9, 158.9, 150.4, 144.4, 135.5, 135.4, 135.3, 130.3, 130.3, 128.3, 128.1, 127.3, 113.5, 113.4, 111.1, 88.5, 87.0, 81.2, 79.2, 75.8, 74.0, 69.7, 61.3, 57.7, 55.4, 51.0, 12.0.

HRMS [ESI^+, MeOH] calculated mass *m/z*: 690.2534 for $C_{36}H_{37}N_5O_8Na$ $[M+Na]^+$; found *m/z*: 690.2537.

ν_{max} 2358 (m, N=N=N), 2108 (m, CΞC), 1687 (s, C=O), 1250 (s, C-O alkyl aryl ether), 828, 702 (s, C-H) cm^{-1}.

5′-*O*-(4,4′-Dimethoxytrityl)-3′-*O*-propargyl-2′-*O*-(2-aminoethyl) thymidine (8)

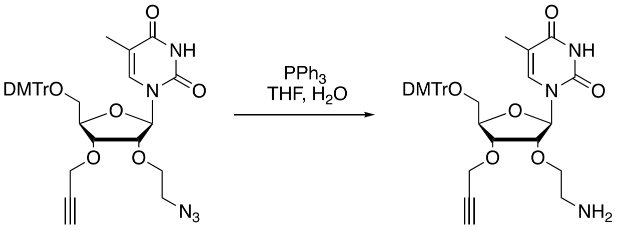

Compound **7** (1.5 g, 2.25 mmol, 1.0 eq) was dissolved in THF (15 mL) and triphenylphosphine (1.2 g, 4.5 mmol, 2.0 eq) was added followed by H_2O (0.2 mL, 11.3 mmol, 5.0 eq). The reaction mixture was stirred for 2 hours at 45 °C. The solvent was then removed *in vacuo* and the oil was purified by flash column chromatography on silica gel (10-60% MeOH in CH_2Cl_2 with constant 1% pyridine) giving compound **8** as a crude white foam (1.12 g) which was used in the next step without further purification.

δ_H (400 MHz, $CDCl_3$) 7.72 (1H, d, *J* = 1.3), 7.45 – 7.40 (2H, m), 7.40 – 7.15 (7H, m), 6.84 (4H, d, *J* = 8.2), 5.97 (1H, d, *J* = 2.9), 4.52 (1H, dd, *J* = 6.9, 5.0), 4.34 – 4.20 (3H, m), 4.16 (1H, dd, *J* = 5.0, 2.9), 3.87 (1H, dt, *J* = 10.2, 5.1), 3.79 (6H, s), 3.70 (1H, dt, *J* = 9.8, 5.1), 3.56 (1H, dd, *J* = 11.1, 2.1), 3.40 (1H, dd, *J* = 11.1, 2.6), 2.92 (2H, app t, *J* = 5.1), 2.43 (1H, t, *J* = 2.4), 1.37 (3H, d, *J* = 1.3).

LRMS [ESI^+, MeCN] calculated mass *m/z*: 664.3 for $C_{36}H_{39}N_3O_8Na$ $[M+Na]^+$; found *m/z*: 664.1

5′-*O*-(4,4′-Dimethoxytrityl)-3′-*O*-propargyl-2′-*O*-(2-(fluorenylmethoxyamido)ethyl)-thymidine (9)

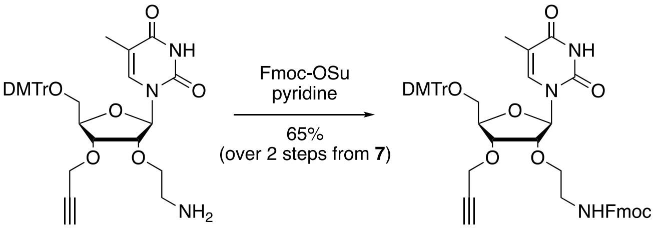

Crude amine **8** (1.0 g, 1.56 mmol, 1.0 eq) was dissolved in anhydrous CH_2Cl_2 (12 mL), followed by addition of pyridine (0.25 mL, 3.1 mmol, 2.0 eq) and then Fmoc-*N*-succinimidyl carbonate (1.8 g. 5.3

mmol, 3.4 eq). The reaction mixture was stirred at room temperature for 2 hours and then was diluted in CH_2Cl_2 and partitioned between sat.aq. NH_4Cl and sat. aq. $NaHCO_3$. The organic layer was dried over Na_2SO_4, filtered and concentrated *in vacuo*. The crude material was purified by flash column chromatography on silica gel (1-3% MeOH in CH_2Cl_2 with constant 1% pyridine) affording compound **9** as a white foam (1.12 g, 1.30 mmol, 65% over 2 steps from **7**).

δ_H (400 MHz, $CDCl_3$) 8.83 (1H, s), 7.78 – 7.66 (3H, m), 7.60 (2H, d, *J* = 7.5), 7.50 – 7.34 (4H, m), 7.35 – 7.08 (9H, m), 6.84 (4H, d, *J* = 7.9), 5.93 (1H, d, *J* = 2.6), 5.50 (1H, t, *J* = 5.7), 4.48 (1H, dd, *J* = 7.1, 5.0), 4.40 (2H, d, *J* = 6.9), 4.27 – 4.01 (5H, m), 3.91 (1H, dt, *J* = 10.1, 5.0), 3.79 (6H, s), 3.80 – 3.71 (1H, m), 3.59 (1H, dd, *J* = 11.1, 2.2), 3.52 – 3.36 (2H, m), 3.38 (1H, dd, *J* = 11.1, 2.6), 2.39 (1H, s), 1.38 (3H, s).

δ_C (101 MHz, $CDCl_3$) 163.8, 158.9, 158.9, 156.6, 150.5, 149.1, 144.4, 144.2, 144.1, 141.4, 136.9, 135.5, 135.4, 135.1, 130.3, 130.2, 128.3, 128.2, 127.8, 127.3, 127.2, 125.2, 120.1, 113.5, 113.4, 111.2, 88.5, 87.0, 81.3, 81.3, 79.1, 76.0, 74.5, 70.2, 66.7, 61.5, 57.8, 55.4, 47.4, 41.1, 12.0.

HRMS [ESI$^+$, MeOH] calculated mass *m/z*: 886.3310 for $C_{51}H_{49}N_3O_{10}Na$ [M+Na]$^+$; found *m/z*: 886.3308

ν_{max} 2161 (w, C≡C), 1687 (s, C=O), 1250 (s, C-O alkyl aryl ether), 758, 741 (s, C-H) cm^{-1}.

2′-Aminoethoxy-*N*-Fmoc-5′-*O*-dimethoxytrityl 3′-thymidinyltriazoyl thymidine (12)

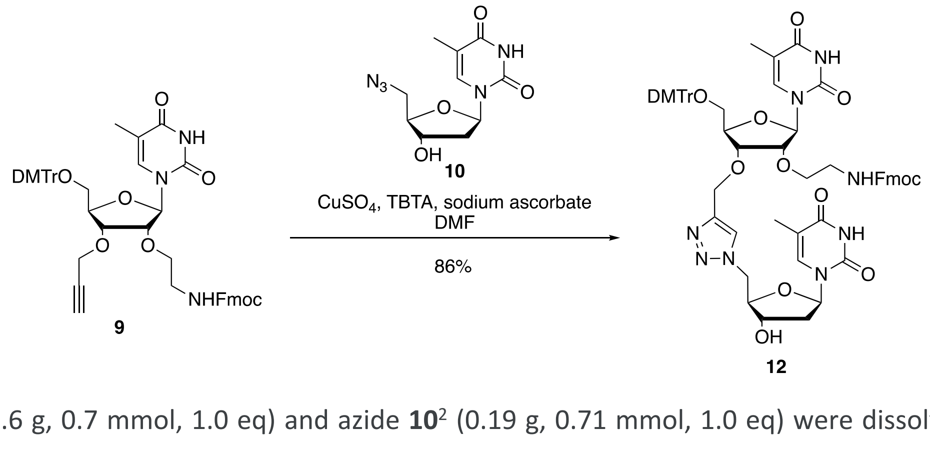

Alkyne **9** (0.6 g, 0.7 mmol, 1.0 eq) and azide **10**[2] (0.19 g, 0.71 mmol, 1.0 eq) were dissolved in DMF (2 mL). Tris(benzyltriazolylmethyl)amine (TBTA) ligand (0.15 g, 0.28 mmol, 0.4 eq) was added followed

by $CuSO_4$ (0.086 g, 0.35 mmol, 0.5 eq) in 0.2 mL of water and sodium ascorbate (0.28, 1.4 mmol, 2 eq) in 0.2 mL of water. The reaction was stirred under argon at room temperature for 4 hours. The solvent was removed *in vacuo*. The residue was dissolved in 50 mL of CH_2Cl_2 (with 5% MeOH) and washed with a 5% aqueous solution of ethylene diaminetetraacetic acid disodium salt (EDTA) (1 x 50 mL) water (1 x 50 mL) and brine (1 x 50 mL). The organic layer was dried over Na_2SO_4 and the solvent was removed *in vacuo*. The crude material was purified by column chromatography on silica gel (5-10% ^{i}PrOH in CH_2Cl_2) yielding compound **12** as a white solid (0.68 g, 0.60 mmol, 86%).

δ_H (400 MHz, DMSO-*d*$_6$) 11.39 (1H, br s), 11.29 (1H, br s), 8.07 (1H, s), 7.86 (2H, d, *J* = 7.6), 7.65 (2H, dd, *J* = 7.7, 2.7), 7.50 (1H, s), 7.45 – 7.11 (14H, m), 6.90 (4H, d, *J* = 8.6), 6.14 (1H, t, *J* = 6.9), 5.84 (1H, d, *J* = 3.9), 5.49 (1H, d, *J* = 4.3), 4.91 – 4.50 (4H, m), 4.40 – 4.35 (1H, m), 4.34 – 4.21 (4H, m), 4.20 – 4.15 (1H, m), 4.12 – 4.01 (2H, m), 3.73 (6H, s), 3.72 – 3.62 (1H, m), 3.57 (1H, dt, *J* = 10.4, 5.7), 3.30 – 3.09 (4H, m), 2.18 (1H, dt, *J* = 13.7, 6.9), 2.12 – 2.05 (1H, m), 1.76 (3H, s), 1.38 (3H, s).

δ_C (101 MHz, DMSO-*d*$_6$) 163.7, 163.6, 158.2, 158.1, 156.2, 150.3, 144.5, 143.9, 143.7, 140.7, 136.0, 135.3, 135.3, 135.0, 129.7, 127.9, 127.6, 127.5, 127.0, 126.8, 125.1, 124.7, 120.0, 113.3, 109.8, 109.5, 86.9, 86.0, 84.1, 83.9, 80.7, 79.5, 75.4, 70.7, 68.6, 65.4, 62.6, 62.3, 55.0, 51.2, 46.7, 39.9 (from DEPT), 37.9, 25.5, 12.0, 11.7.

HRMS [ESI$^+$, MeOH] calculated mass *m/z*: 1153.42777 for $C_{61}H_{62}N_8O_{14}Na$ [M+Na]$^+$; found *m/z*: 1153.42480

ν_{max} 2962 (w, C-H alkane), 1687 (s, C=O), 1251 (s, C-O alkyl aryl ether), 1078 (s, C-O secondary alcohol), 791, 742 cm$^{-1.}$

2′-Aminoethoxy-*N*-Fmoc-5′-*O*-dimethoxytrityl 3′-thymidinyltriazoyl benzoylcytidine (13)

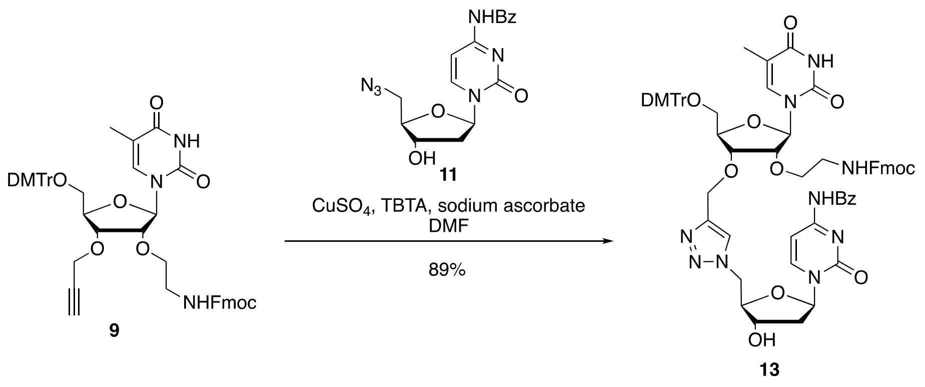

Alkyne **9** (0.79 g, 0.91 mmol, 1 eq) and azide **11**[3] (0.32 g, 0.90 mmol, 1.0 eq) monomers were dissolved in DMF (10 mL). TBTA ligand (0.20 g, 0.37 mmol, 0.4 eq) was added followed by addition of $CuSO_4$ (0.14 g, 0.88 mmol, 1 eq) in 0.5 mL of water and sodium ascorbate (0.44 g, 2.22 mmol, 2.5 eq) in 0.5 mL of water. The reaction was stirred under argon at room temperature for 4 hours. Upon completion the solvent was removed *in vacuo*. The residue was dissolved in CH_2Cl_2 (with 5% MeOH) and washed with a 5% aqueous EDTA solution (50 mL) then washed with H_2O (50 mL) and brine (50 mL). The organic layer was dried over Na_2SO_4 and evaporated to dryness. The crude material was purified by column chromatography on silica gel (5-10% ^{i}PrOH in CH_2Cl_2) to give a white solid (0.98 g) as a 1:0.4 mixture of compound **13**:TBTA ligand.

δ_H (600 MHz, DMSO-d_6) 11.40 (1H, s), 11.23 (1H, s), 8.15 (1H, s), 8.08 (1.2H, s, TBTA), 8.06 (1H, d, *J* = 7.5), 7.99 (2H, d, *J* = 7.2), 7.85 (2H, d, *J* = 7.5), 7.66 – 7.59 (3H, m), 7.54 – 7.46 (3H, m), 7.42 – 7.20 (21H, m, contains TBTA peaks), 6.96 – 6.83 (4H, m), 6.10 (1H, t, *J* = 6.4), 5.84 (1H, d, *J* = 4.0), 5.59 (2.4H, s, TBTA), 5.55 (1H, d, *J* = 4.4), 4.75 – 4.60 (4H, m), 4.40 (1H, t, *J* = 5.4), 4.30 (1H, t, *J* = 4.5), 4.28 – 4.21 (3H, m), 4.21 – 4.14 (2H, m), 4.14 – 4.08 (1H, m), 3.72 (6H, s), 3.70 – 3.64 (1H, m), 3.62 (2.4H, s, TBTA), 3.61 – 3.55 (1H, m), 3.26 (1H, dd, *J* = 11.0, 3.6), 3.24 – 3.10 (3H, m), 2.36 – 2.23 (1H, m), 2.17 (1H, dt, *J* = 13.1, 6.4), 1.36 (3H, s).

δ_C (151 MHz, DMSO-d_6) 167.3, 163.7, 163.1, 162.3, 158.2, 158.1, 156.2, 154.2, 150.3, 145.1, 144.5, 143.9, 143.7, 143.7, 140.7, 136.2, 135.3, 135.2, 135.0, 133.1, 132.7, 129.7, 128.7, 128.4, 128.0, 127.9,

127.7, 127.6, 127.5, 127.0, 126.8, 125.1, 124.8, 124.2 (TBTA), 120.0, 113.3, 109.4, 96.3, 86.9, 86.5, 86.0, 84.8, 80.7, 79.5, 75.3, 70.7, 68.6, 65.4, 62.6, 62.2, 55.0, 54.9, 52.7 (TBTA), 51.3, 46.9 (TBTA), 46.7, 40.1, 40.6, 11.7. (Includes 4 unassigned TBTA peaks)

HRMS [ESI$^+$, MeOH] calculated mass *m/z*: 1220.4724 for $C_{67}H_{66}N_9O_{14}$ [M+H]$^+$; found *m/z*: 1220.4707

2′-Aminoethoxy-*N*-Fmoc-5′-*O*-dimethoxytrityl-3′-thymidynyltriazoyl thymidine phosphoramidite(1)

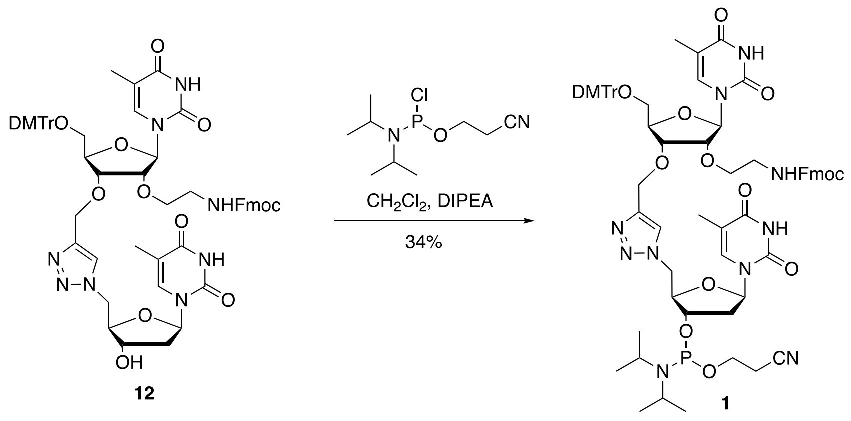

Dimer **12** (0.60 g, 0.53 mmol, 1.0 eq) was co-evaporated with dry pyridine, then dissolved in anhydrous CH_2Cl_2 (8 mL) and kept under argon. *N,N*-Diisopropylethylamine (DIPEA) (0.19 mL, 1.1 mmol, 2.1 eq) was added to the solution, followed by slow addition of 2-cyanoethyl-*N,N*-diisopropylchlorophosphoramidite (0.21 mL, 0.94 mmol, 1.8 eq) at 0 °C. The reaction was allowed to stir under argon at room temperature for 4 hours. The reaction mixture was diluted with degassed CH_2Cl_2 (20 mL), washed with degassed sat. aq. KCl solution (25 mL), dried over Na_2SO_4 and concentrated *in vacuo*. The crude material was purified by column chromatography on silica gel under argon (50-100% ethyl acetate in CH_2Cl_2 with 0.5% pyridine) affording compound **1** as a white solid (0.22 g, 0.17 mmol, 34%).

δ_H (400 MHz, Acetonitrile-*d*$_3$) 9.05 (1H, s), 8.99 (1H, s), 7.83 – 7.69 (2H, m), 7.61 (2H, t, *J* = 6.6), 7.48 (1H, s), 7.50 – 7.20 (14H, m), 6.97 (1H, d, *J* = 13.0), 6.88 (4H, d, *J* = 8.5), 6.19 – 6.13 (1H, m), 6.10 – 6.01 (1H, m), 5.83 (1H, d, *J* = 3.2), 4.71 – 4.47 (5H, m), 4.41 – 4.03 (6H, m), 3.85 – 3.54 (6H, m), 3.76 (6H, s),

3.37 – 3.21 (4H, m), 2.64 (2H, t, *J* = 5.9), 2.45 – 2.18 (2H, m), 1.77 (3H, s), 1.41 (3H, s), 1.26 – 1.13 (12H, m).

δ_P (162 MHz, Acetonitrile-d_3) δ 148.86 (s), 148.63 (s)

2′-Aminoethoxy-*N*-Fmoc-5′-*O*-dimethoxytrityl-3′-thymidynyltriazoyl benzoylcytidine phosphoramidite (2)

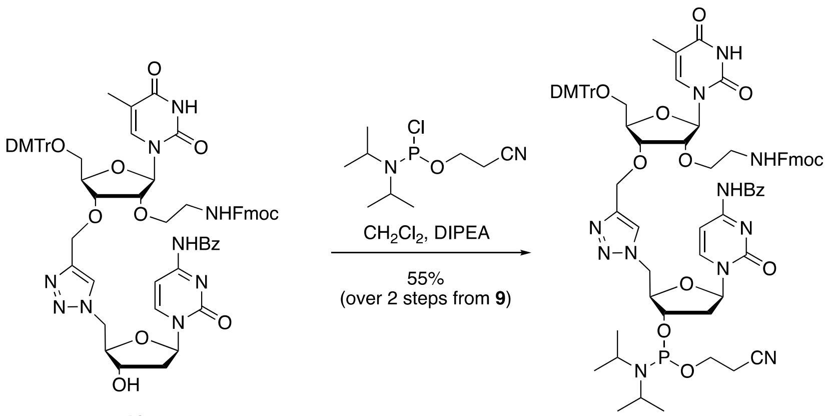

Dimer **13** (0.760 g, 0.623 mmol, 1.0 eq) was co-evaporated with dry pyridine, then dissolved in anhydrous CH_2Cl_2 (6 mL) under argon. DIPEA (0.245 mL, 1.4 mmol, 2.2 eq) was added to the solution, followed by slow addition of 2-cyanoethyl-*N,N*-diisopropylchlorophosphoramidite (0.240 mL, 1.08 mmol, 1.7 eq) at 0 ^oC. The reaction was stirred under argon at room temperature for 4 hours. The reaction mixture was diluted with deoxygenated CH_2Cl_2 (20 mL) and washed with deoxygenated sat. aq. KCl solution (25 mL). The organic layer was dried over sodium sulfate and concentrated *in vacuo*. The crude material was purified by column chromatography on a silica gel under argon (20% hexane in CH_2Cl_2) affording product **2** as a white foam (0.557 g, 0.39 mmol, 63%).

δ_H (400 MHz, Acetonitrile-d_3) δ = 8.96 (1H, br s), 7.91 (2H, d, *J* = 7.8), 7.87 – 7.69 (3H, m), 7.64 – 7.59 (4H, m), 7.52 – 7.46 (3H, m), 7.44 – 7.11 (15H, m), 6.96 – 6.79 (4H, m), 6.26 – 6.19 (1H, m), 6.04 (1H, q, *J* = 6.3), 5.84 (1H, d, *J* = 3.3), 4.75 – 4.50 (5H, m), 4.44 – 4.10 (7H, m), 3.84 – 3.56 (12H, m), 3.30 (4H, d, *J* = 4.6), 2.73 – 2.56 (2H, m), 2.55 – 2.41 (1H, m), 2.30 – 2.18 (1H, m), 1.41 (3H, d, *J* = 1.1), 1.37 – 1.04 (12H, m).

δ_P (162 MHz, Acetonitrile-d_3) 150.39 (s), 150.08 (s)

1-(Methylamino)anthraquinone-4-[(6-*O*-(4-nitrophenyl)-hexyl)amino carbonate (4)

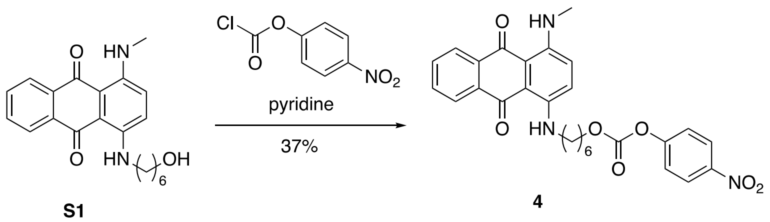

Compound **S1** [4, 5] (0.05 g, 0.14 mmol, 1.0 eq) was dissolved in anhydrous CH_2Cl_2 (7 mL), followed by addition of 4-nitrophenyl chloroformate (0.057 g, 0.28 mmol, 2.0 eq) and anhydrous pyridine (0.057 mL, 0.70 mmol, 5.0 eq). The reaction was stirred for 6 hours at room temperature. The solvent was removed *in vacuo.* The crude mixture was dissolved in CH_2Cl_2 (20 mL) and washed with H_2O and brine. The organic layer was dried over Na_2SO_4 and the crude material was purified by column chromatography on silica gel (20-40% ethyl acetate in petroleum ether) to give compound **4** (0.045 g, 0.052 mmol) as dark blue solid in 37% yield.

δ_H (600 MHz, $CDCl_3$) 10.65 (1H, t, J = 5.3), 10.52 (1H, app d, J = 5.2), 8.23 – 8.19 (2H, m), 8.14 (2H, d, J = 9.2), 7.72 – 7.48 (2H, m), 7.28 – 7.22 (2H, m), 7.15 – 7.08 (2H, m), 4.19 (2H, t, J = 6.6), 3.30 (2H, td, J = 7.0, 5.4), 2.98 (3H, d, J = 5.2), 1.73 – 1.64 (4H, m), 1.50 – 1.35 (4H, m).

δ_C (151 MHz, $CDCl_3$) 182.6, 182.5, 155.7, 152.7, 147.1, 146.2, 145.5, 134.6, 134.6, 132.2, (2 resonances), 126.2, 126.1, 125.4, 123.6, 123.2, 121.9, 110.1, 109.9, 69.6, 42.9, 29.7, 29.6, 28.6, 26.9, 25.7.

HRMS [ESI$^+$, MeOH] calculated mass m/z: 518.1922 for $C_{28}H_{27}N_3O_7$ $[M+H]^+$; found m/z: 518.1923

Oligonucleotide synthesis

General

DNA reagents including standard DNA and RNA phosphoramidites and solid supports (controlled pore glass (CPG) resins) were purchased from Link Technologies Ltd, Glen Research and GE Healthcare. Oligonucleotides were synthesised on an Applied Biosystems 394 automated DNA/RNA synthesiser or ÄKTA oligopilot plus. 2'-OMe-RNA cyanoethyl (CE) phosphoramidites were purchased from Link Technologies and used for all phosphorothioate oligonucleotide sequences. Phenylacetyl disulphide (PADS) or 3-ethoxy-1,2,4-dithiazoline-5-one (EDITH) reagents were used for the sulfurisation step. Coupling efficiencies and overall oligonucleotide yields were determined by the automated trityl cation conductivity monitoring facility of the synthesiser and were ≥98.0%. Standard and modified phosphoramidite monomers were dissolved in anhydrous acetonitrile to a concentration of 0.1 M immediately prior to use. The coupling time for dA, dG, dC and dT monomers, was set to 60 s for standard monomers and up to 12 min for modified ones. The coupling time for 2'-OMe-RNA CE monomers, was set to 360 s. All oligonucleotides were characterised by negative-mode electrospray HPLC-mass spectrometry, using XEVO G2-QTOF MS instrument or Bruker micrOTOF mass spectrometer and an Acquity UPLC system with a BEH C18 1.7 µm column (Waters). Raw data were processed and deconvoluted using MaxEnt or the Data Analysis function of the Bruker Daltronics Compass™ 1.3 software package.

Unmodified DNA strands

DNA synthesis was performed on 1.0 µmole scale using standard phosphoramidite chemistry involving cycles of acid catalyzed detritylation, coupling, capping, and iodine oxidation. Standard DNA phosphoramidites were coupled for 60 s. Coupling efficiencies were ≥98.0% in all cases. Cleavage of the oligonucleotides from the solid support and deprotection was achieved by exposure to concentrated aqueous ammonia solution for 60 min at room temperature followed by heating in a sealed tube for 5 h at 55 °C. Oligonucleotides were purified by RP-HPLC using a Gilson HPLC system with ABI Aquapore C8 column (8 mm x 250 mm, pore size 300 Å) using a gradient of 0 to 50% MeCN

in 0.1M triethylammonium bicarbonate (TEAB) at pH 7.5 over 20 min with a flow rate of 4 mL/min. The fractions from HPLC were evaporated without additional desalting.

Unmodified RNA strands

RNA synthesis was performed on an Applied Biosystems 394 automated DNA/RNA synthesiser using a standard phosphoramidite cycle of detritylation, coupling, capping, and oxidation on a 1.0 µmole scale. SynBase™CPG 1000/110 (Link Technologies) and were packed into a twist column (Glen research) for synthesis. 2′-O-TBDMS RNA phosphoramidites A-tac, C-tac, G-tac and U, where tac=tert-butylphenoxyacetyl (Sigma-Aldrich) were dissolved in anhydrous acetonitrile (0.1 M) immediately prior to use. Coupling, capping and oxidation reagents were 5-benzylthio-1H-tetrazole (0.3 M in acetonitrile; Link Technologies), fast deprotection Cap A (5%tert-butylphenoxyacetyl acetic anhydride in tetrahydrofuran)/Cap B (16%N-methylimidazole in tetrahydrofuran) and iodine (0.1 M in tetrahydrofuran, pyridine and water), respectively. The coupling time during RNA synthesis was 10 min. Stepwise coupling efficiencies were determined by automated trityl cation conductivity monitoring and in all cases were >97%.

Cleavage of the RNA from the solid support and deprotection was achieved by exposure to concentrated aqueous ammonia solution for 2.5 at room temperature followed by heating in a sealed tube for 2.5 h at 55 °C. Ammonia was removed in vacuo, and the solution was freeze-dried, re-dissolved in a 1:1 mixture of dry DMSO (300 µL) and triethylaminetrihydrofluoride (300 µL) and heated for 2.5 h at 65 °C. After cooling down to room temperature, sodium acetate (3 M pH 5.2, 50 µL) and butanol (3 mL) were added and the RNA was stored for minimum of 30 min at−80 °C. The RNA was then pelleted by centrifugation (12,000 ×g, 30 min, 4 °C), the supernatant discarded and washed twice with 70% ethanol (750 µL) then dried in vacuo. The pellet was dissolved in water and purified by RP-HPLC using a Gilson HPLC system with ABI Aquapore C8 column (8 mm x 250 mm, pore size 300 Å) using a gradient of 0 to 50% MeCN in 0.1M triethylammonium bicarbonate (TEAB) at pH 7.5 over 20 min with a flow rate of 4 mL/min. The fractions from HPLC were evaporated without additional desalting.

Synthesis and purification of phosphodiester modified oligonucleotides using phosphoramidites 1 and 2.

2´-Aminoethoxy modified oligonucleotides were synthesised using automated solid-phase oligonucleotide synthesis on a 1.0 µmol scale involving cycles of acid-catalysed detritylation, coupling, capping, and iodine oxidation using an Applied Biosystems 394 synthesiser. Standard DNA phosphoramidites were coupled for 60 s, whereas extended coupling time of 10 min was used for the triazole dimer phosphoramidites **1** and **2** and the 5′-*O*-DMT-2′-aminoethoxythymidine-3′-*O*-phosphoramidite[1]. Coupling efficiencies were ≥98.0% as determined by the inbuilt automated trityl cation conductivity monitoring facility. Following removal of the 4,4′-dimethoxytrityl group, the oligonucleotides were kept on the solid support. The resin was treated with diethylamine (10% in acetonitrile) for 20 min in order to selectively remove the cyanoethyl groups. Next, oligonucleotides were cleaved from the solid support (CPG resin) and deprotected by suspending the resin in concentrated aqueous ammonia followed by heating in a sealed tube for 5 h at 55 °C. Oligonucleotides were purified by RP-HPLC using a Gilson HPLC system with ABI Aquapore C8 column (8 mm x 250 mm, pore size 300 Å) using a gradient of 0 to 50% MeCN in 0.1M triethylammonium bicarbonate (TEAB) at pH 7.5 over 20 min with a flow rate of 4 mL/min. The fractions from HPLC were evaporated without additional desalting.

Synthesis and purification of phosphorothioate modified oligonucleotides containing triazole linkages and 2′-aminoethoxy groups

Oligonucleotide synthesis was performed on 1.0 µmole scale using standard phosphoramidite chemistry involving cycles of acid catalysed detritylation, coupling, capping, and sulfurisation (using either PADS or EDITH in accordance with manufacturers guidelines). 2'-OMe-RNA CE phosphoramidites were coupled for 360 s and for the modified monomer T-T triazole dimer **1**, T-C triazole dimer **2** and 2′-aminoethoxy T[1] coupling times were extended to 12 min. After synthesis, oligonucleotides were kept on the solid support. Coupling efficiencies were ≥98.0%. Modified oligonucleotides on resin were treated with a solution of diethylamine (10% in acetonitryl) for 20 min in order to selectively remove the cyanoethyl protecting groups. Next, oligonucleotides were cleaved

from the solid support (CPG resin) and deprotected by suspending the resin in concentrated aqueous ammonia followed by heating in a sealed tube for 5 h at 55 °C. Oligonucleotides were purified by RP-HPLC using a Gilson HPLC system with Gemini-NX 10u C18 column (250 mm x 21.2 mm, pore size 110 Å). The phosphorothioate oligonucleotides were purified using a gradient of 15 to 40% acetonitrile in 0.1 M hexylammonium acetate (HAA) pH 8.0. The fractions from HPLC were evaporated and desalted by gel filtration using NAP-25 and NAP-10 columns (GE Healthcare).

Post synthetic functionalisation of modified oligonucleotides

Functionalisation with pyrene 3 or anthraquinone 4

Modified oligonucleotides with the 2′-aminoethoxy group were dissolved in 0.5 M $NaHCO_3$/Na_2CO_3 buffer at pH 8.75 (0.5 mM). 1-Pyrenebutyric acid N-hydroxysuccinimide ester **3** (~ 100 equivalents) or Anthraquinone carbonate **4** was dissolved in DMF in 1:1 ratio with the buffer and added to the oligonucleotide solution. The reaction mixture was then heated at 55 °C for 5 h. The crude mixture was desalted using NAP-25 column and purified by RP-HPLC using a Gilson HPLC system equipped with an ABI Aquapore C8 column (8 mm x 250 mm, pore size 300 Å) for phosphodiester oligonucleotides or a Gemini-NX 10u C18 column (250 mm x 21.2 mm, pore size 110 Å) for phosphorothioate oligonucleotides. A gradient of 0 to 60% acetonitrile in 0.1 M triethylammonium bicarbonate (TEAB) pH 7.5 was used (0 to 90% buffer B over 30 min, flow rate 4 mL/min). The fractions from HPLC were evaporated and used without additional desalting.

Functionalisation with guanidine

The modified oligonucleotide with the 2′-aminoethoxy group was dissolved in H_2O (0.5 mM). 1*H*-pyrazole-1-carboxamidine hydrochloride (PCH) reagent (~ 300 equivalents) and imidazole (~ 10 equivalents) were added and the reaction mixture was heated at 55 °C for 5 h. The crude mixture was desalted using NAP-25 column and purified by RP-HPLC using a Gilson HPLC system with ABI Aquapore C8 column (8 mm x 250 mm, pore size 300 Å) using a gradient of 0 to 50% MeCN in 0.1 M triethylammonium bicarbonate (TEAB) at pH 7.5 over 20 min with a flow rate of 4 mL/min. The fractions from HPLC were evaporated without additional desalting.

Biophysical analysis

UV melting studies

UV melting analysis was performed using a Varian Cary 4000 Scan UV-Visible spectrophotometer. The oligonucleotides (2 or 4 µM, as specified in the text) were dissolved in 10 mM phosphate buffer containing 200 mM NaCl at pH 7.2 and denatured by heating to 85 °C (10 °C/min). The samples were then cooled to 20 °C and reheated to 85 °C at a rate of 1 °C/min, recording the absorbance of the sample at 260 nm as a function of temperature. This was repeated three times and the melting temperature (T_m) calculated by determining the maxima of the 1st derivative of the melting curve using in-built software. All UV melting studies were performed in duplicate with errors of less than ±0.5 °C

Circular dichroism

Circular dichroism spectra were recorded on a Jasco J-720 spectropolarimeter using 4 µM of the duplex in 10 mM phosphate buffer, 200 mM NaCl at pH 7.2. Spectra were recorded between 200-320 nm at a rate of 100 nm/min, with step resolution of 0.2 nm, bandwidth of 1.0 nm and sensitivity of 50 mdeg. Three successive spectra were recorded and an average was taken. A blank (buffer) baseline was subtracted from each spectrum. Spectra were smoothed using the Savitzky-Golay filter function in Origin.

Cell biology

General conditions

All reagents were purchased from Invitrogen, ThermoFisher and Promega, unless stated otherwise.

Comparing ASO activity using Hela pLuc705 cells.

Hela pLuc705 cells were grown in Dulbecco's modified Eagle's medium (DMEM) containing 10% Fetal Bovine Serum (FBS), incubated at 37 °C, 5% CO_2. For transfection experiments, 30,000 cells/well were

seeded in forty-eight well plates with a final media volume of 250 µL/well and transfected the following day using Lipofectamine 2000 (LF2000, Thermofisher) according to manufacturer's protocols. The oligonucleotides were mixed with LF2000 using 1 µL of LF2000 to 1 µg of oligonucleotide in 50 µL of OptiMEM, which was incubated for 20 min in room temperature before cell treatment. The complexes were then added to the cells in 200 µL of OptiMEM and the cells were were incubated for 4 h at 37 °C, 5% CO_2. After 4 h the OptiMEM was replaced with 250 µL of DMEM growth media containing 10% FBS and the cells were incubated for additional 18 h at 37 °C, 5% CO_2 after which the cells were lysed and the luciferase expression measured using the Bright-Glo™ Luciferase Assay System (Promega) according to the manufacturer's instructions. The cells were washed with PBS and lysed using Glo Lysis Buffer (Promega) with gentle agitation for 5 min. This cell lysate (50 µL) was then pipetted to a black 96 well plate, the luciferase assay reagent (50 µL) was added and mixed gently for 2 min. The luminescence generated was recorded over 1 second using a 1420 Victor plate reader.

Cells were treated with the transfection mixture at seven oligonucleotides concentrations: 15.6, 31.3, 62.5, 93.75, 125 and 180 nM.

Treatment of MCF7 cells with oligonucleotides

MCF7 cells (BD BioCat™ 117994) were grown in T 225cm^2 flasks without collagen coating (BD Falcon 353138) with RPMI 1640 media (Invitrogen) containing 10% FBS, 1% GlutaMAX and 1% sodium pyruvate. Cells were split twice in 4 days distance using 0.25% Trypsin. For the plasmid transduction, cells were harvested using 0.25% Trypsin and re-suspended in antibiotic-free media. Cells were counted and their viability determined using ViCell. MCF7 cells were mixed with 15% BacMam (C654T-B-globin intron 2/luciferase pHTBV1) and diluted to 300,000 cells/mL with growth media. 100 µL/well of cell transduction slurry was dispensed into ninety-six well collagen-coated white plates (BD BioCoat) to yield a plating density of 30,000 cells/well. Plates were then centrifuged at 1000 rpm for 1 h and then placed in the incubator for 24 h at 37 °C, 5% CO_2 prior to treatment with oligonucleotides.

After 24 h media containing BacMam was aspirated from MCF7 cells and replaced with 100 µL/well of growth media. For transfection-mediated delivery experiments the transfection mixture was prepared by addition of oligonucleotide with Lipofectamine 2000 in a 1:1 wt:wt ratio in OptiMEM, which was incubated for 20 min in room temperature before the cell treatment. Cells were treated with the transfection mixture with the following oligonucleotide concentrations: 91 pM, 0.27 nM, 0.82 nM, 2.5 nM, 7.4 nM, and 22.2 nM in 100 µL of media. For gymnotic delivery experiments cells were treated with oligonucleotides without transfecting agent at the following concentrations: 0.56, 1.67, 5, 15. 45 µM.

All plates were incubated for 24 h at 37 °C, 5% CO_2. The Steady-Glo® Luciferase Assay System (Promega) was then used to determine luciferase expression. The assay was performed according to the manufacturer's instructions. 50 µL of the luciferase reagent was added to each well and plates were incubated for 10 min at room temperature. The luminescence generated was recorded over 1 seconds using a Synergy H4 plate reader.

NMR spectra of compounds

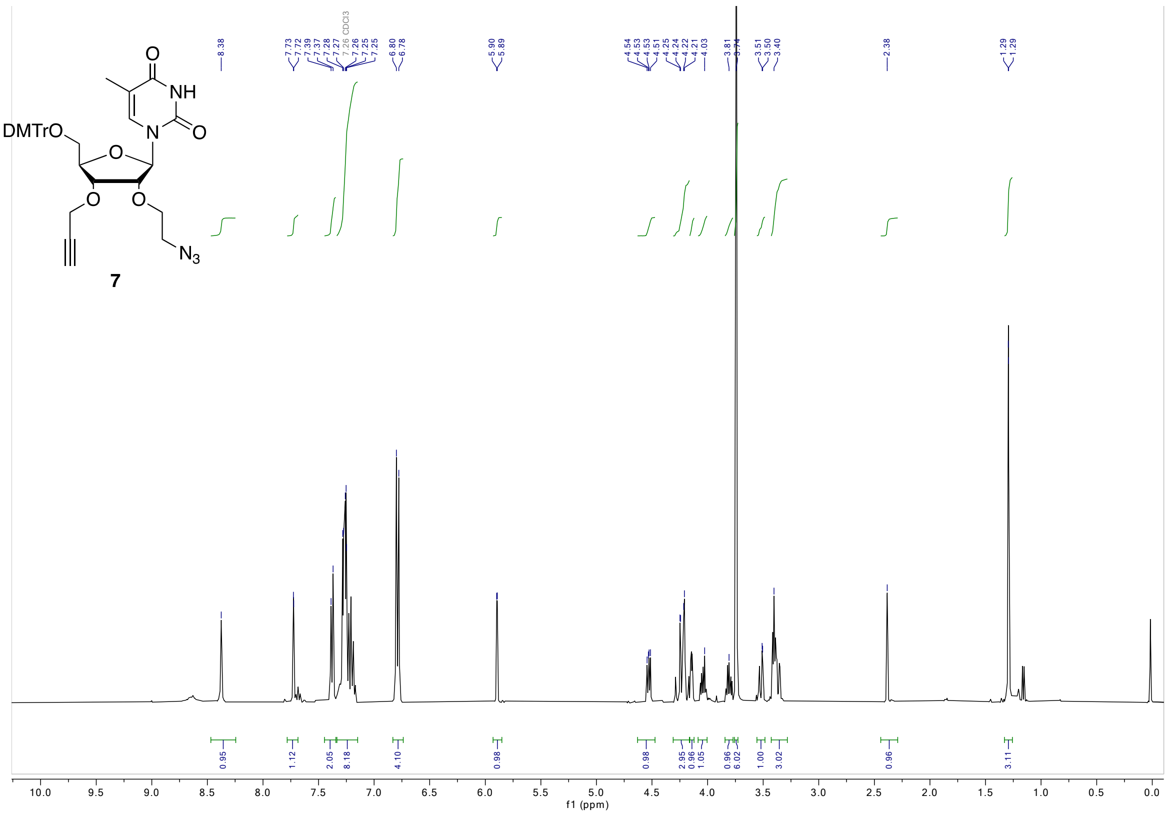

^{1}H NMR spectrum of compound **7**

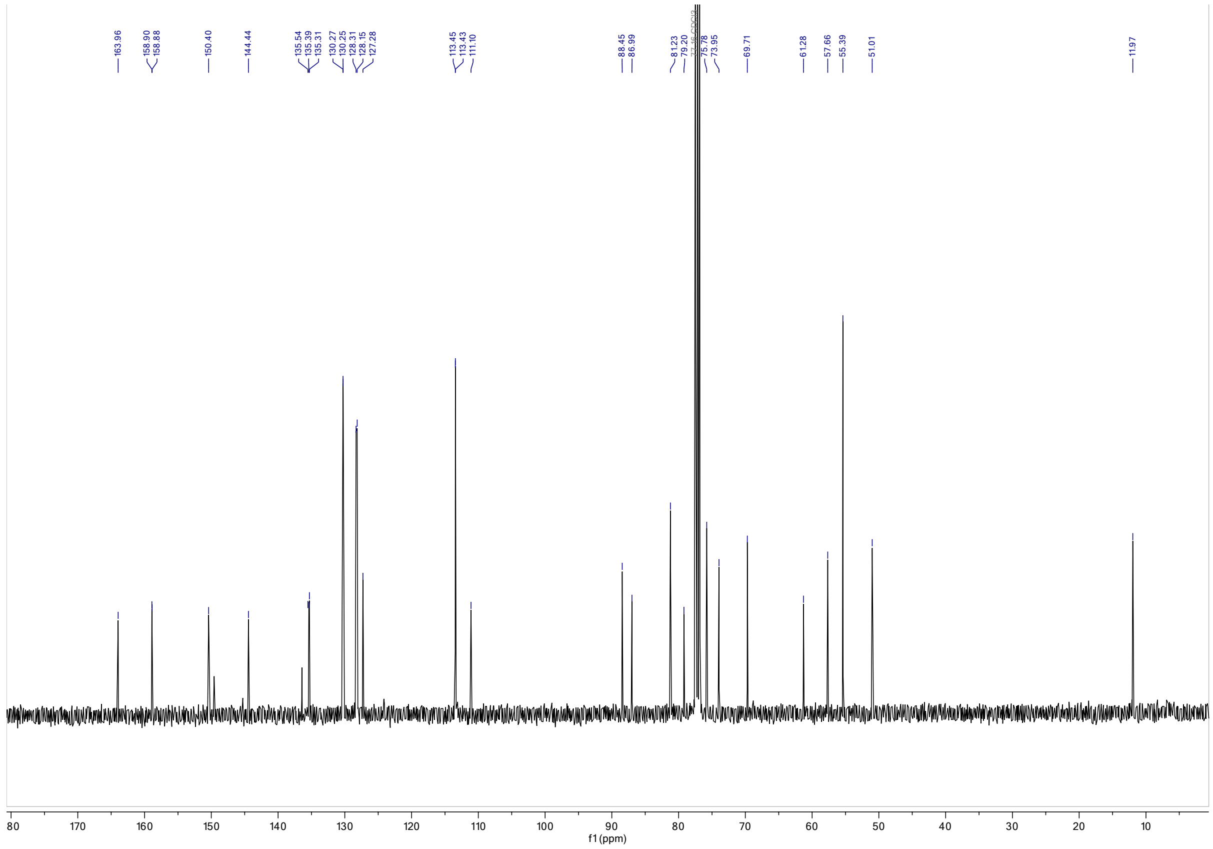

^{13}C NMR spectrum of compound **7**. Solvent peaks: pyridine (149.6 and 136.5)

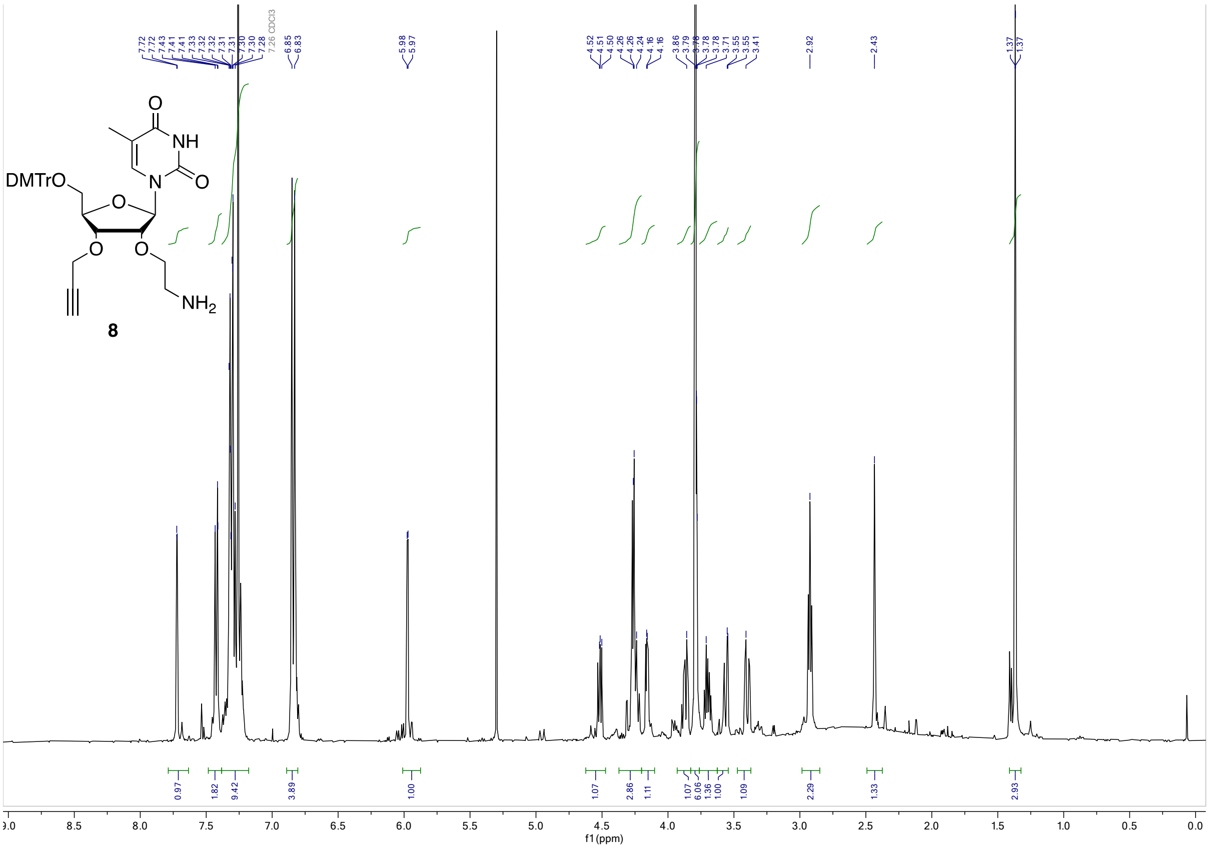

^{1}H NMR spectrum of impure compound **8**.

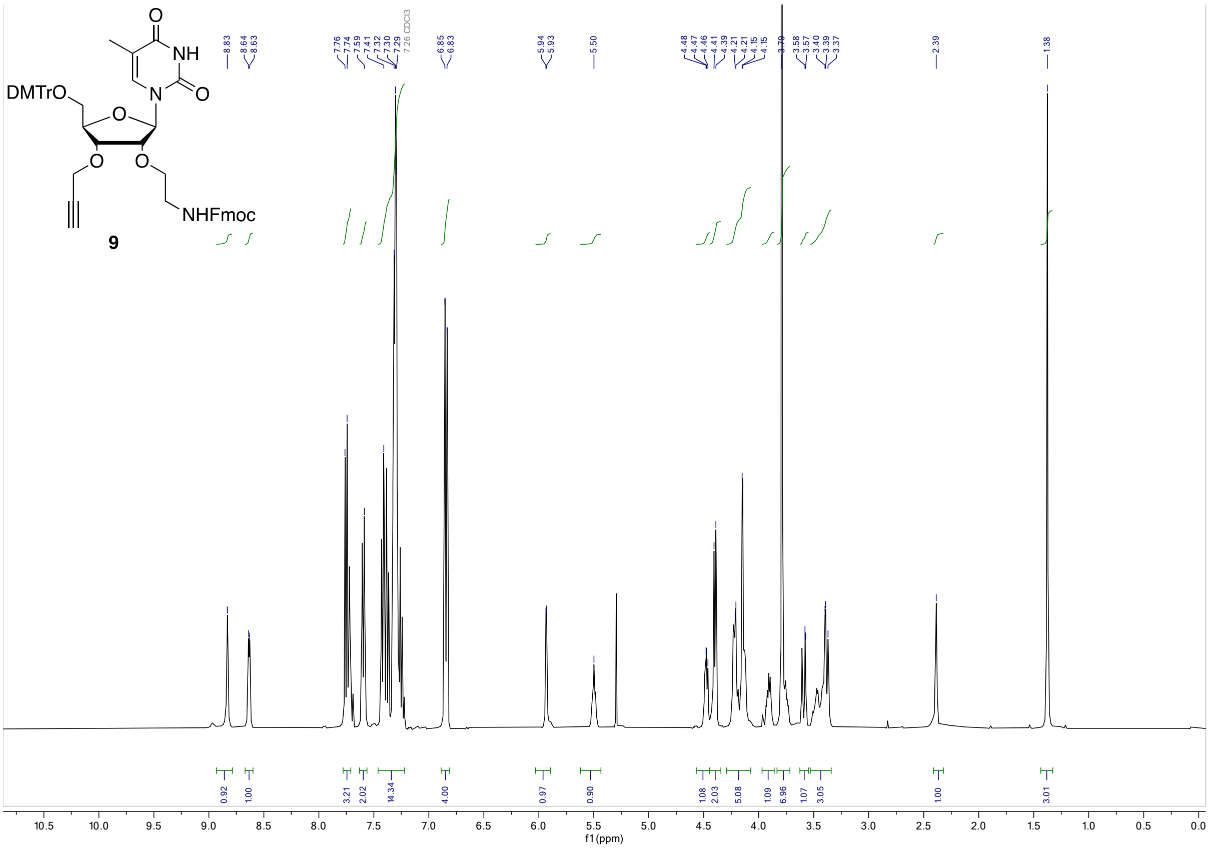

^{1}H NMR spectrum of compound **9** Solvent peaks: pyridine (8.63) and CH_2Cl_2 (5.3)

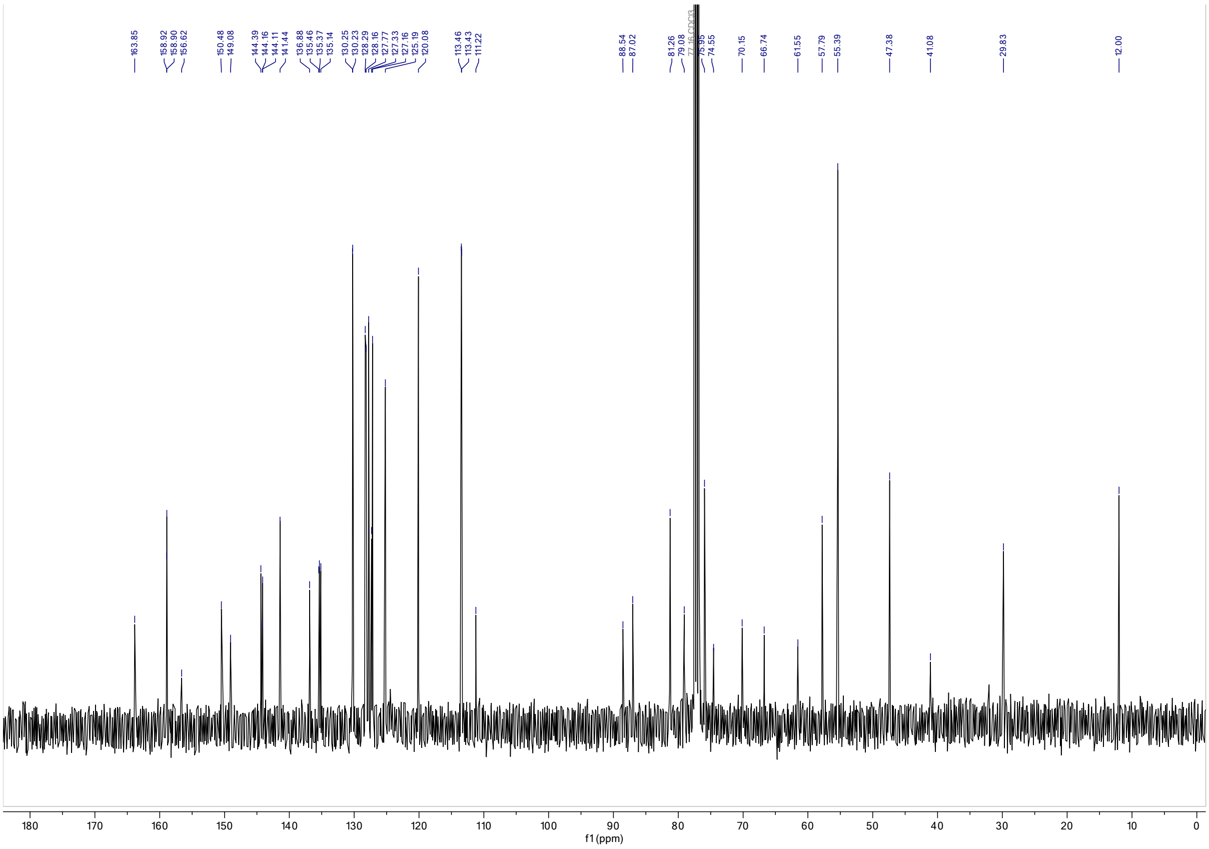

^{13}C NMR spectrum of compound **9**

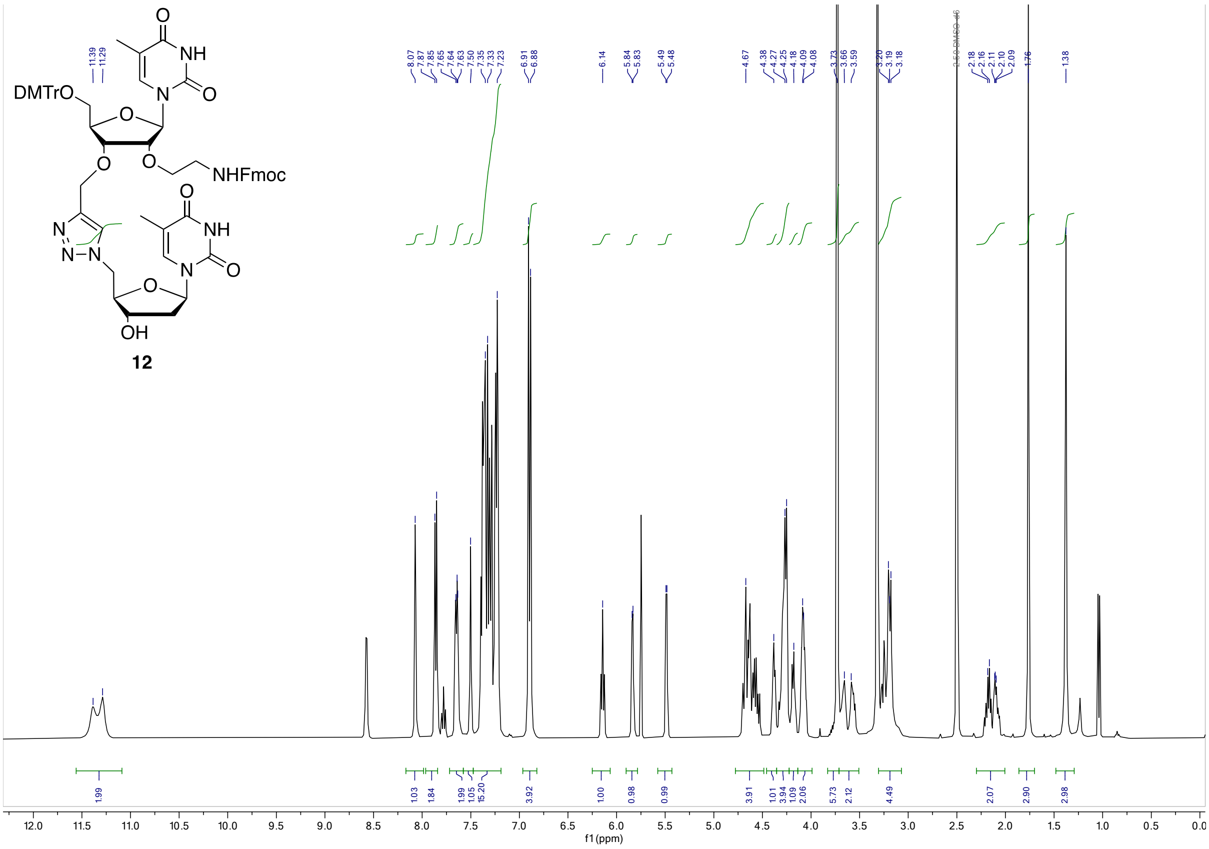

^{1}H NMR of compound **12**. Solvent peaks pyridine (8.58, 7.78), CH_2Cl_2 (5.76) and $^{i}PrOH$ (1.04)

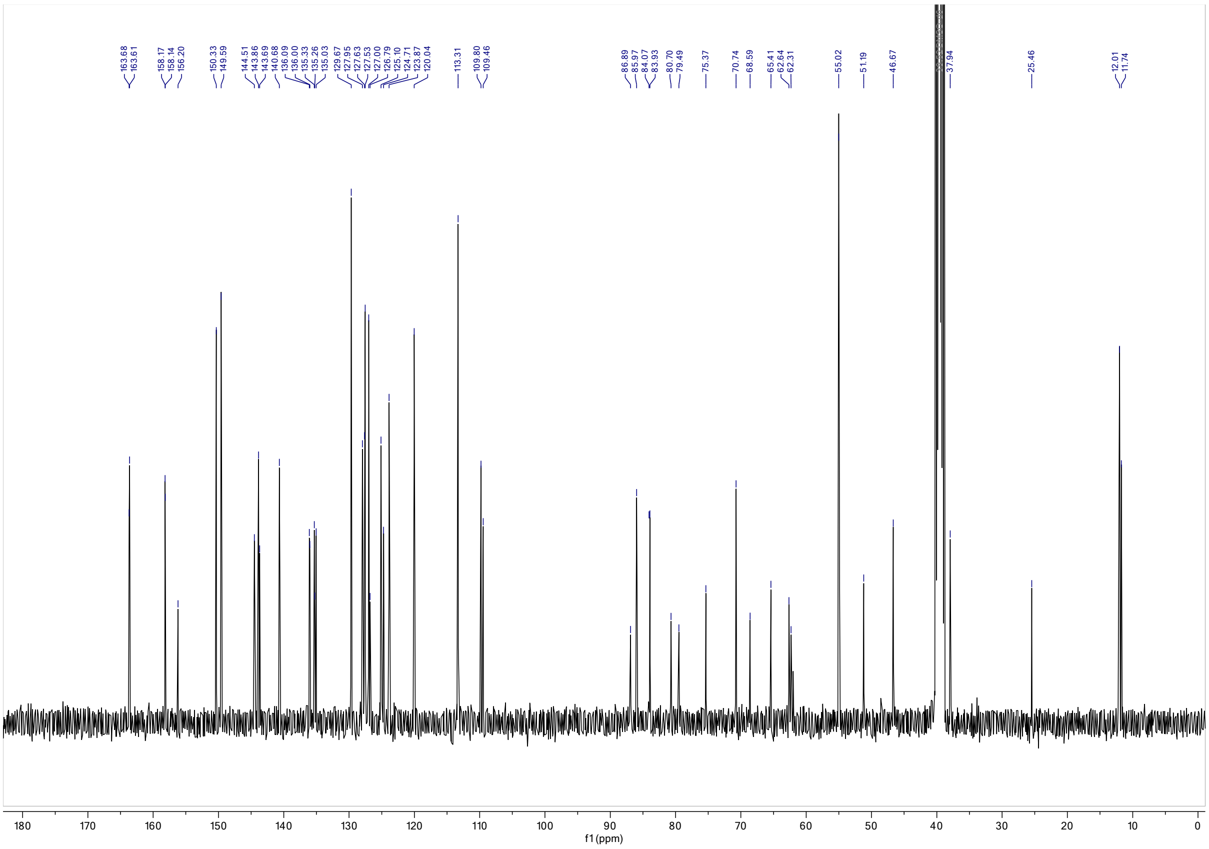

^{13}C NMR of compound **12**. Solvent peaks: pyridine (149.6, 136.1 and 123.9)

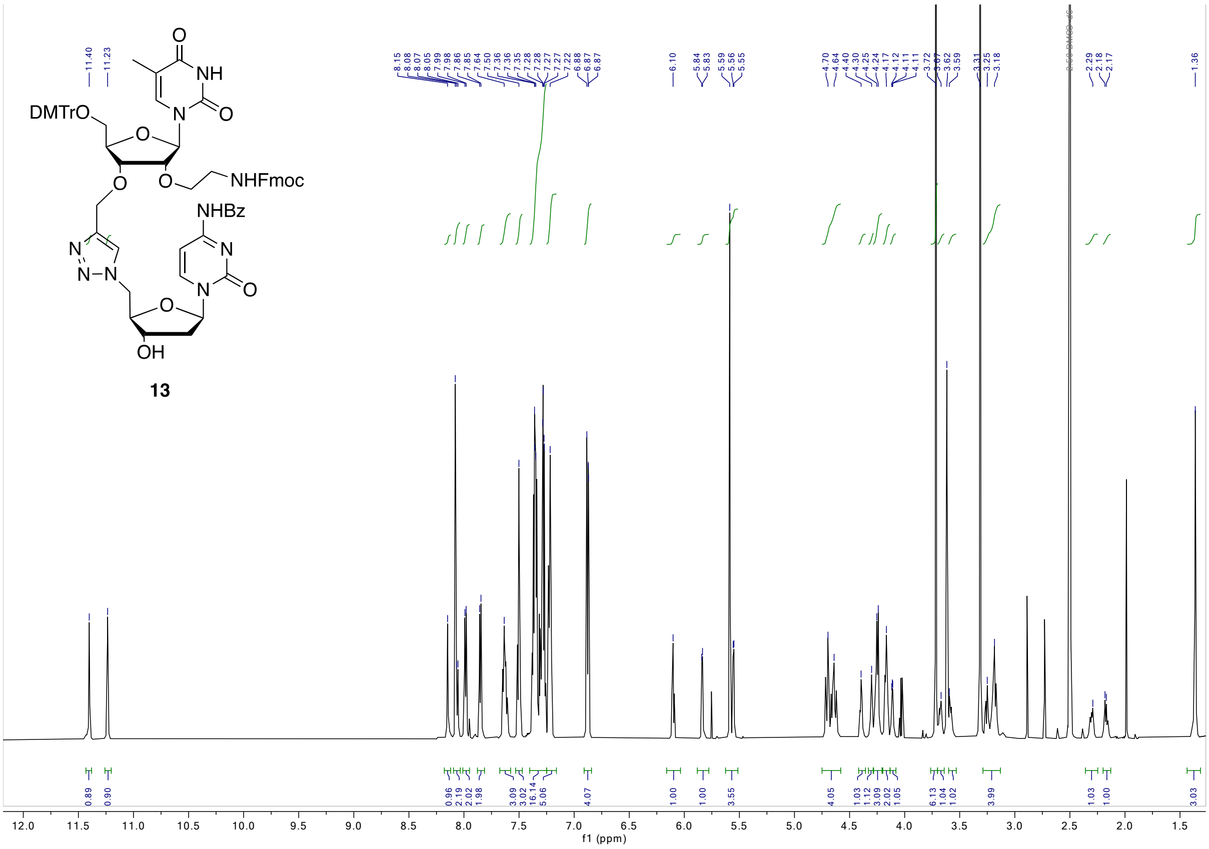

^{1}H NMR of compound **13** with 0.4 equivalents of TBTA ligand

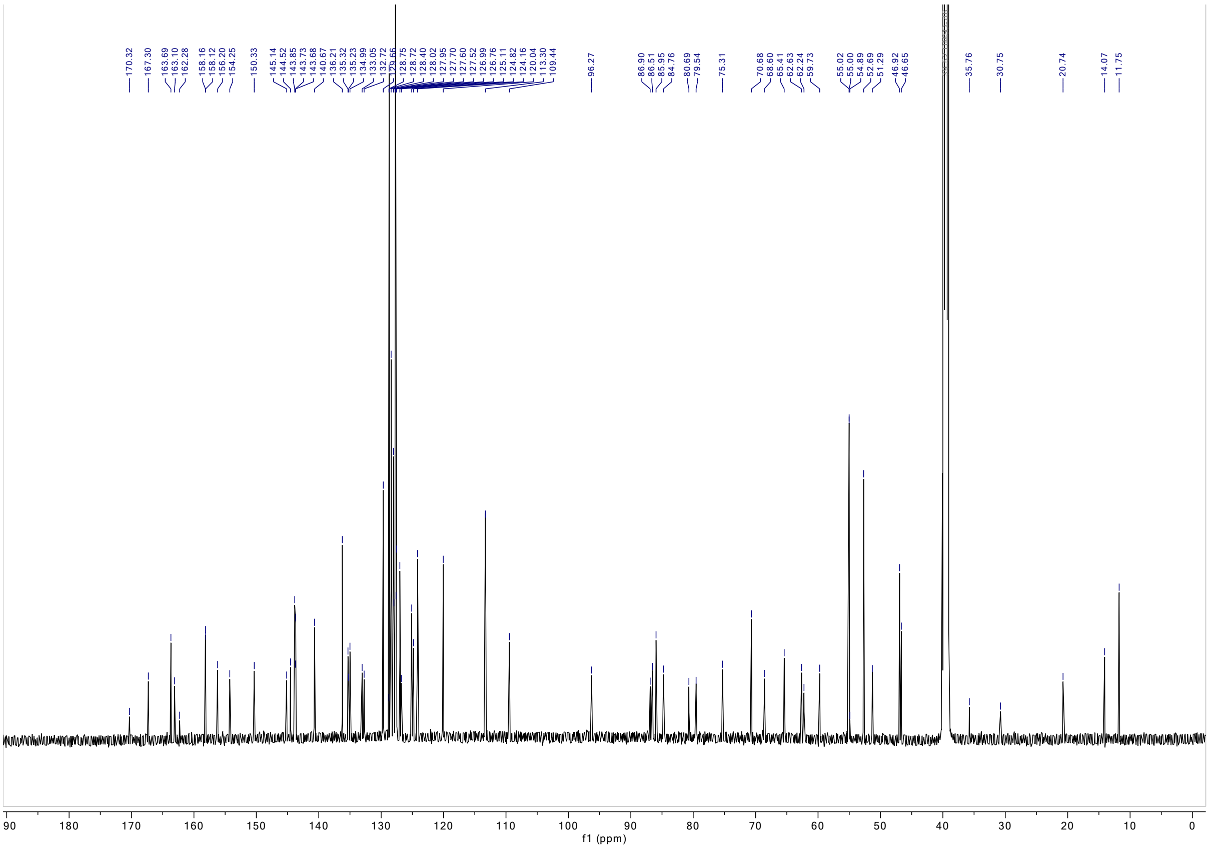

^{13}C NMR of compound **13** with 0.4 equivalents of TBTA ligand

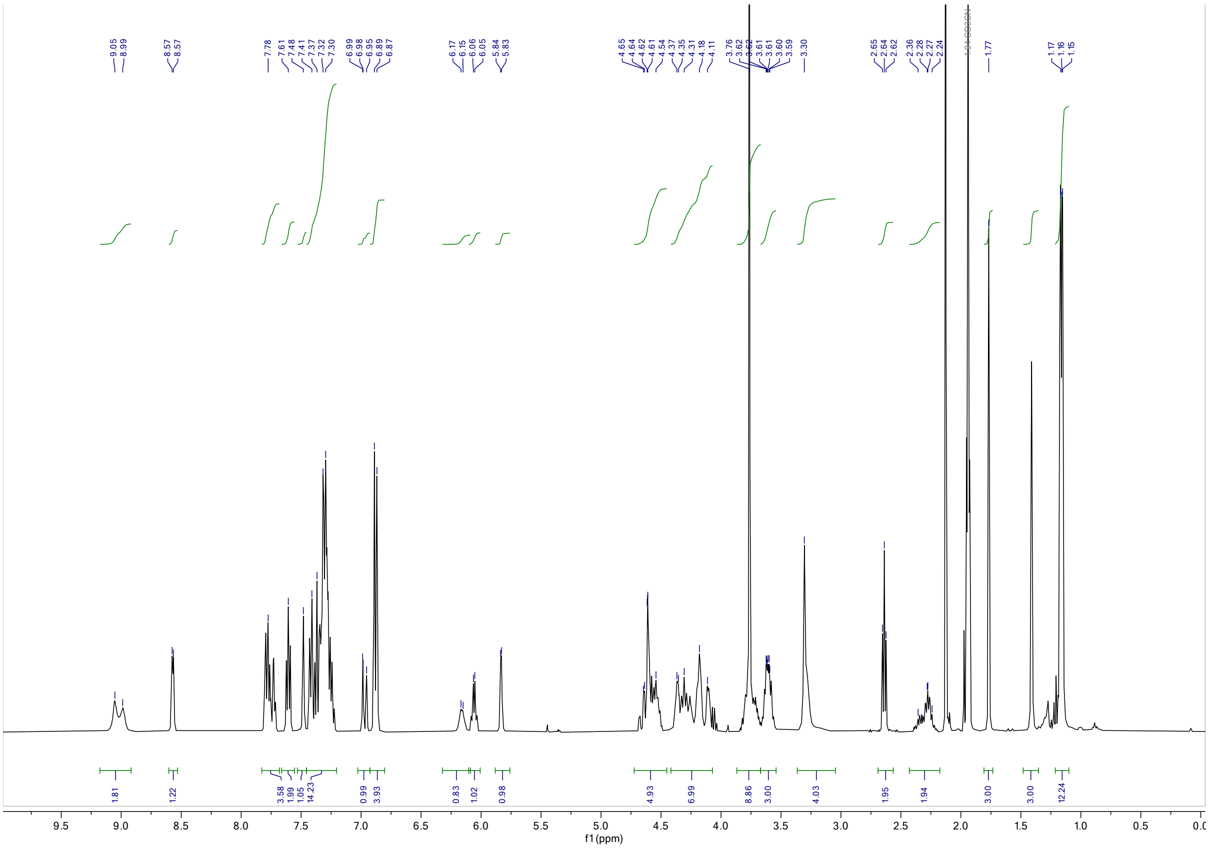

^{1}H NMR of compound **1**. Solvent impurities pyridine (8.57, 7.72, 7.33) and EtOAc (4.06, 1.97, 1.20)

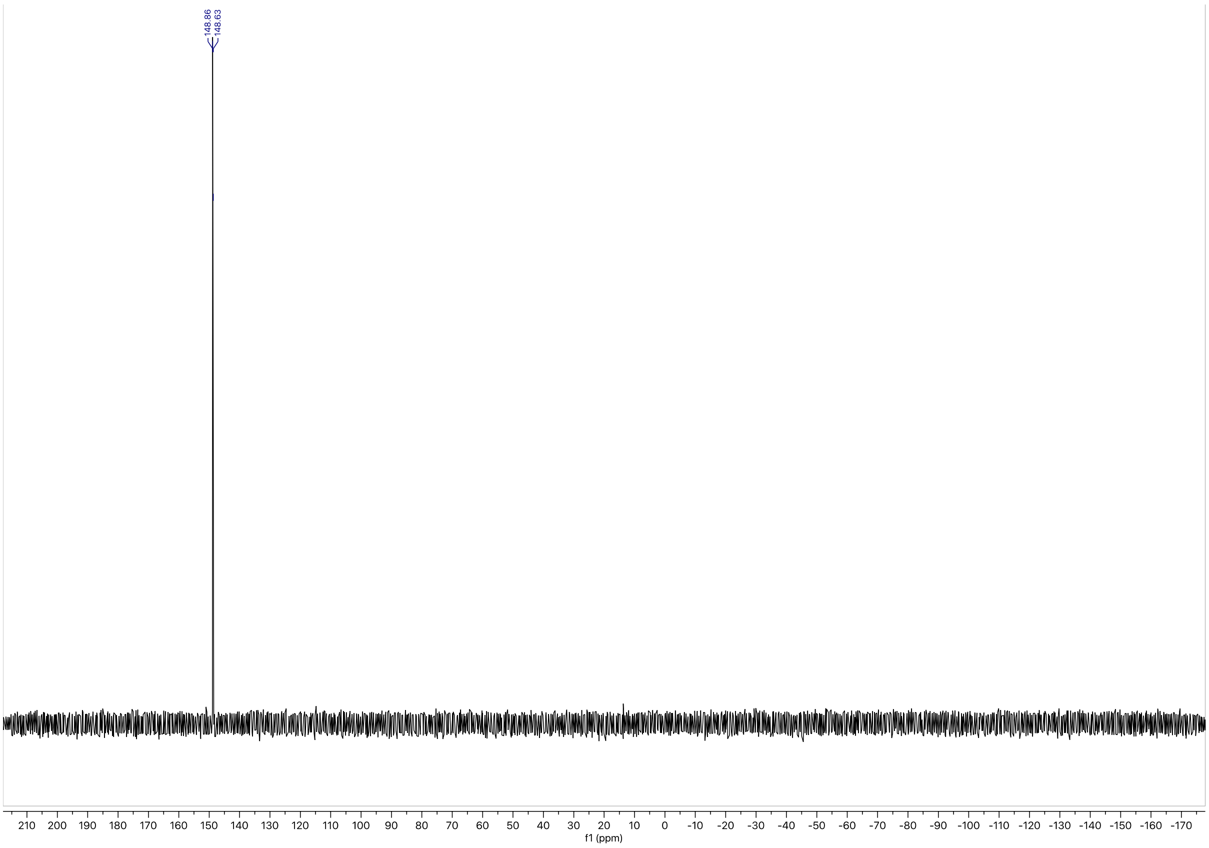

^{31}P NMR of compound **1**

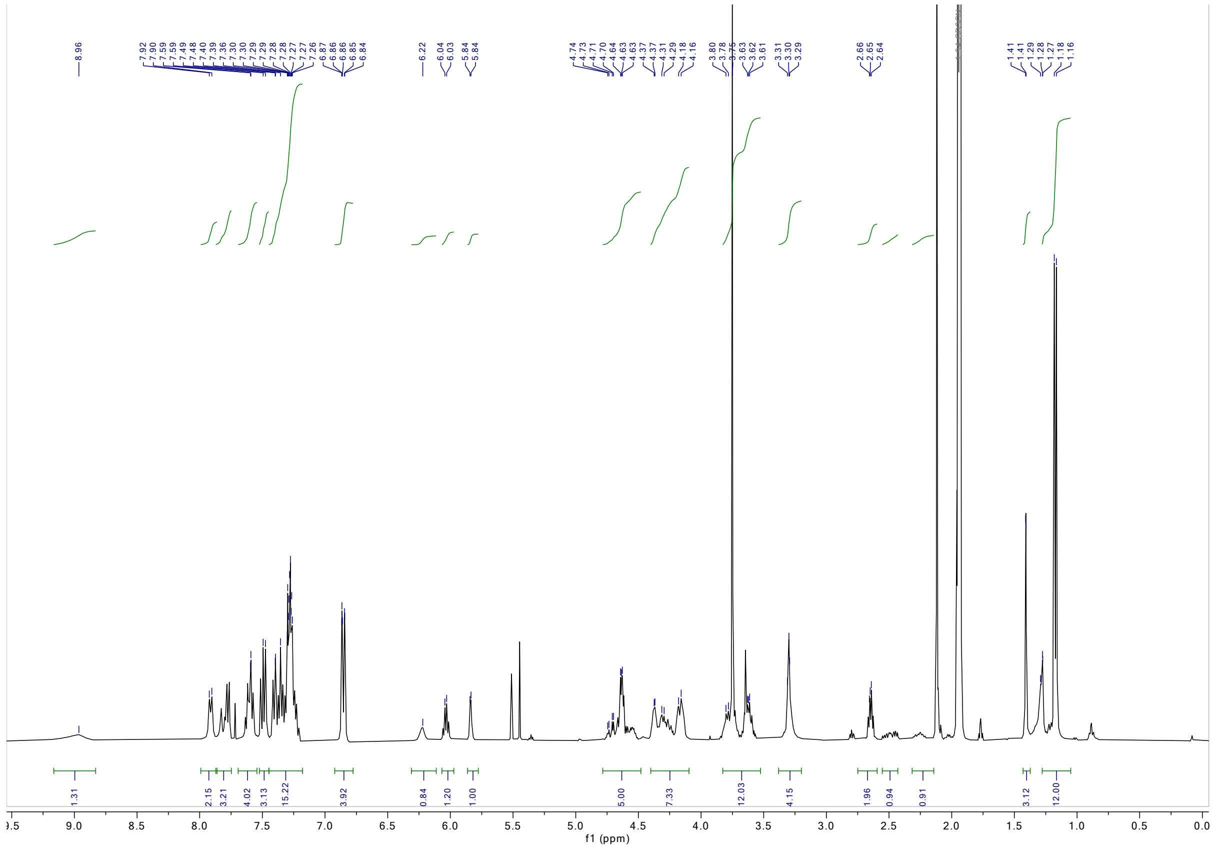

^{1}H NMR of compound **2**

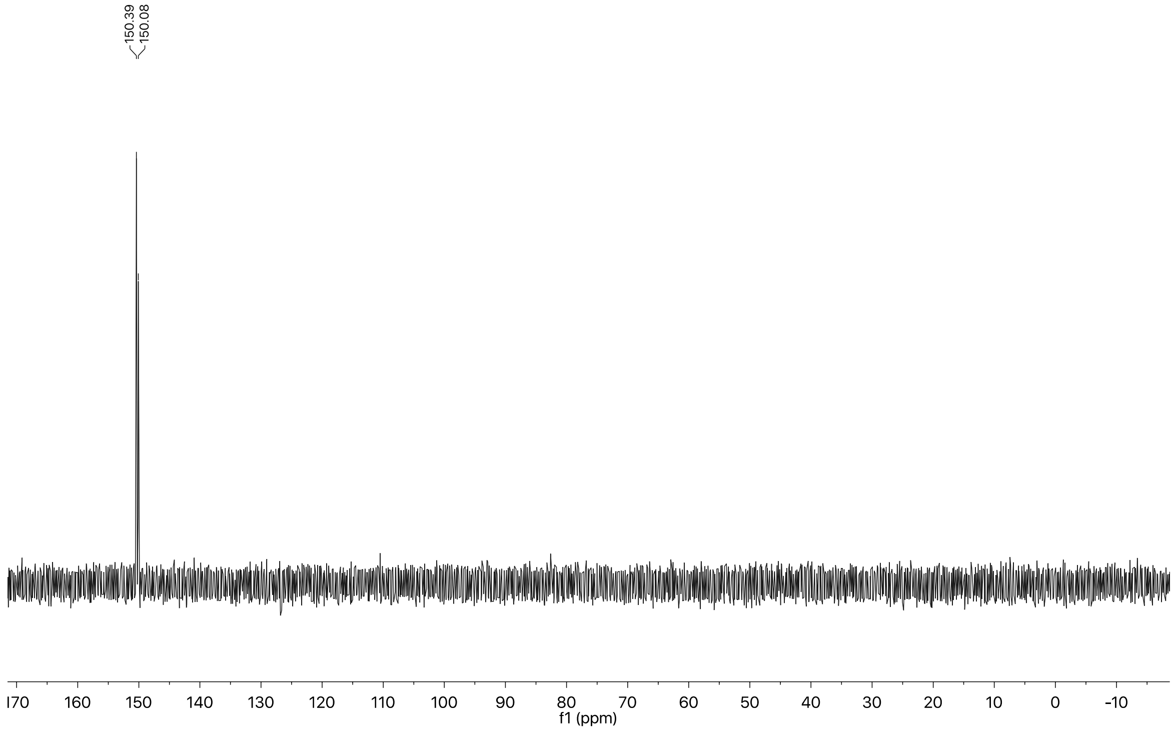

^{31}P NMR of compound **2**

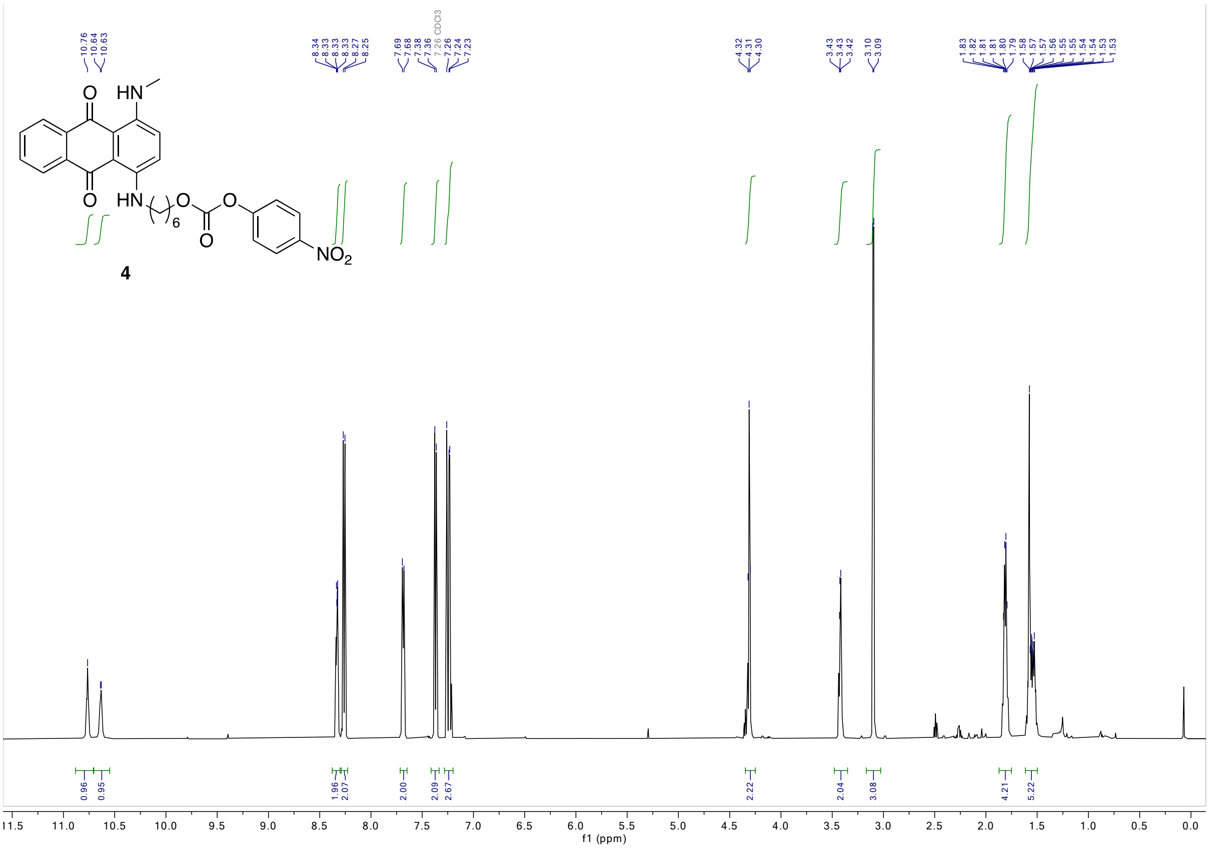

^{1}H NMR of compound **4**

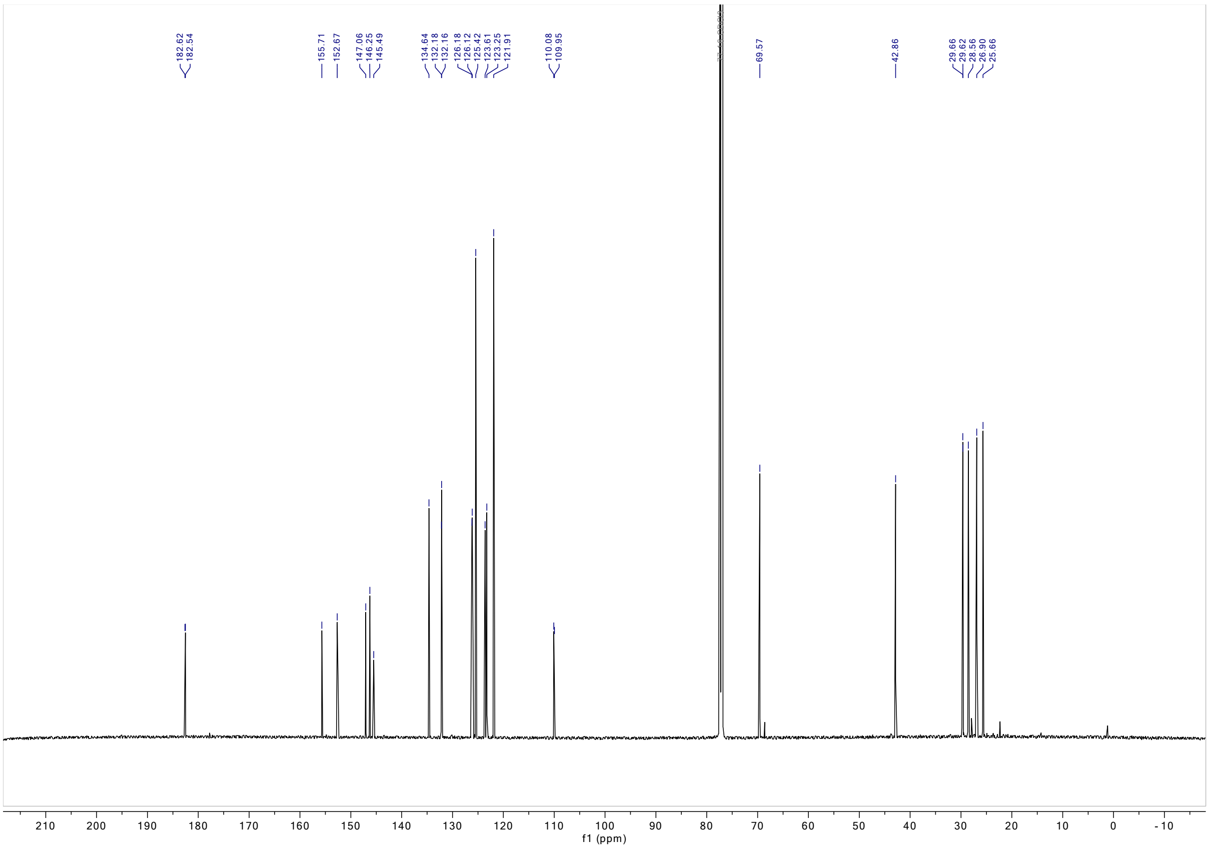

^{13}C NMR of compound **4**

Representative HPLC-mass spectra of oligonucleotides

ON2: CGACGCX^{PY}_{t}TGCAGC

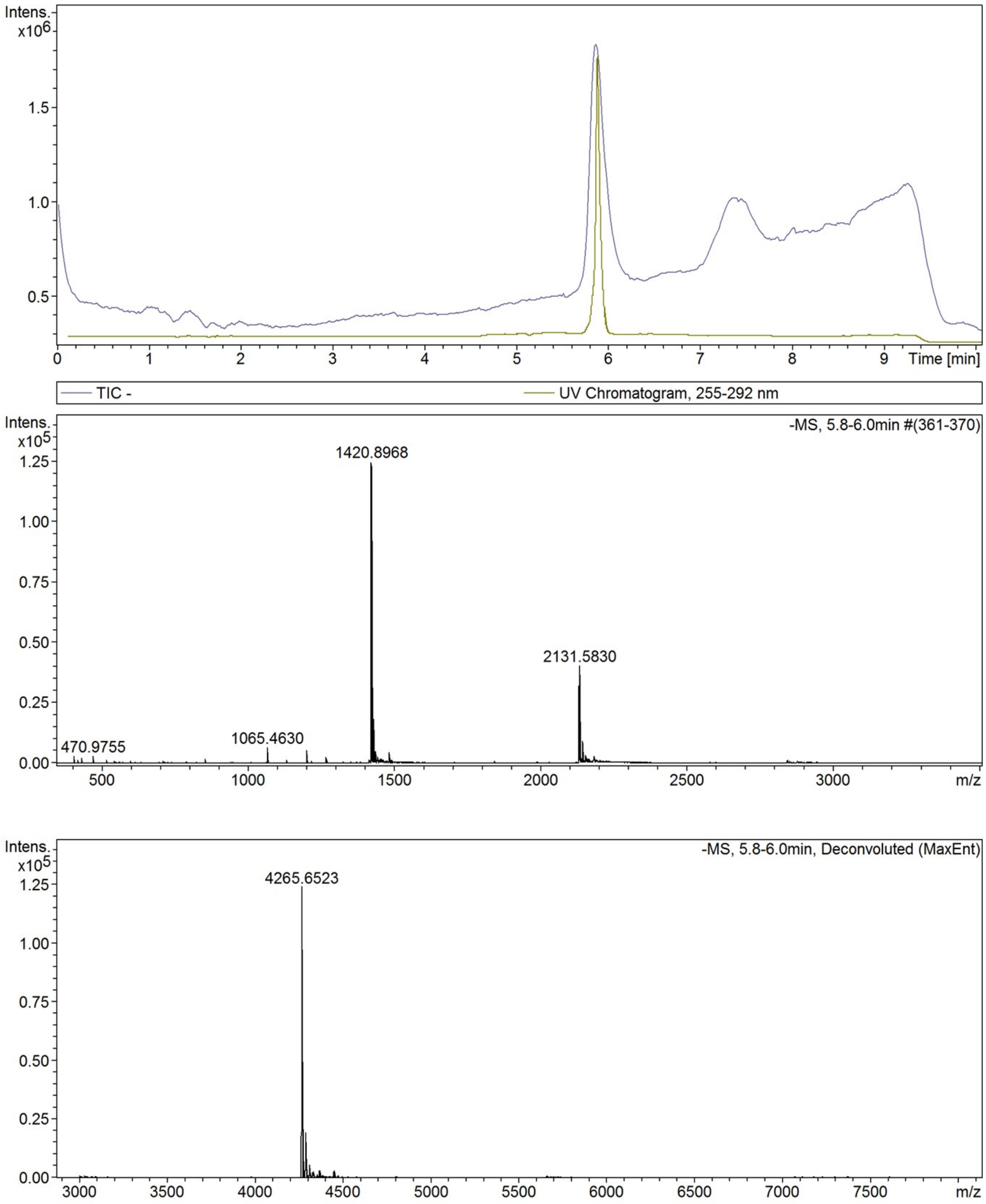

ON13: CCX$^{PY}{}_{t}$CUUACCUCAGUUACA

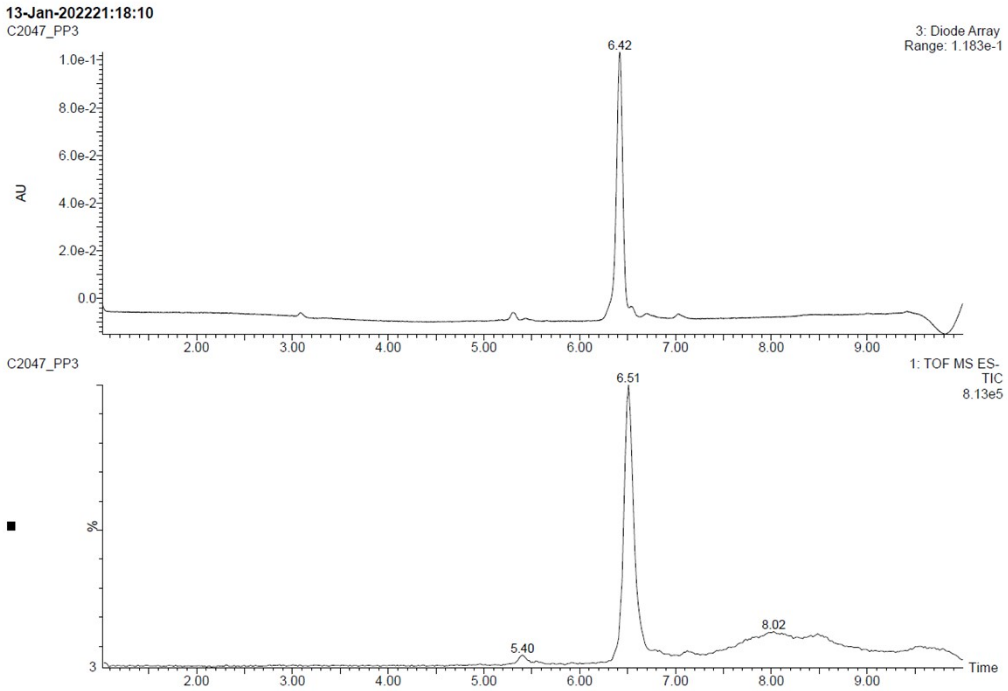

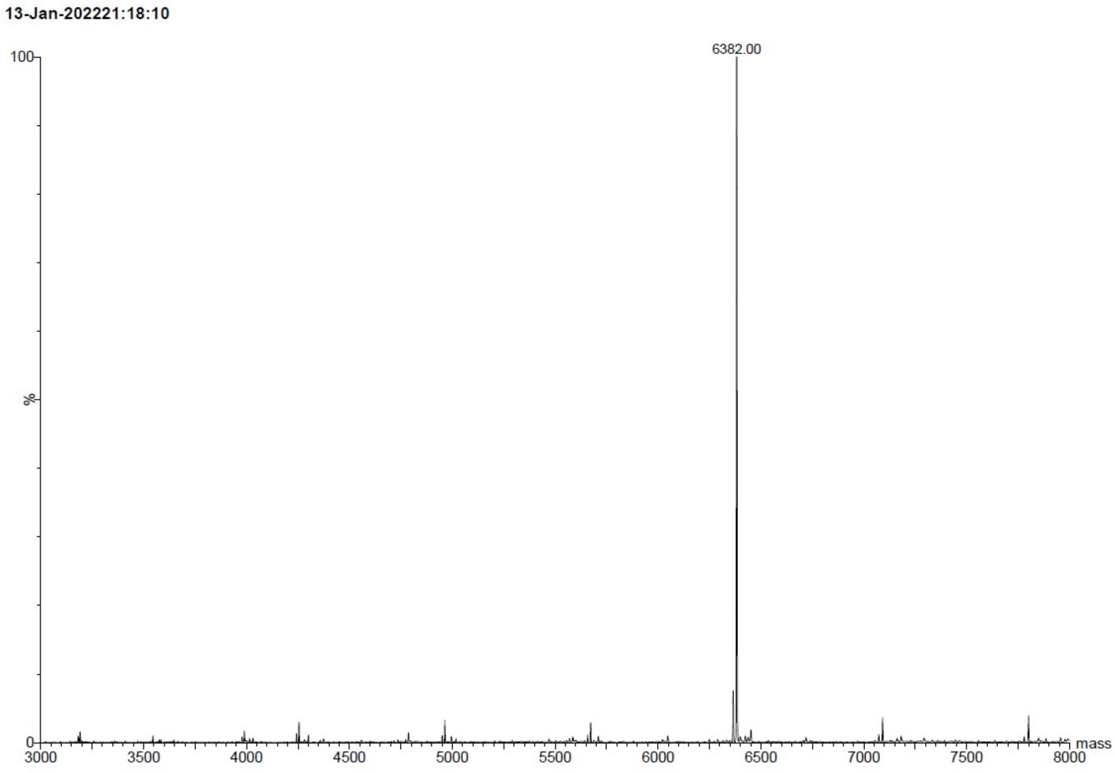

ON21: GCUAX_tTACCX_tTAACCCAG

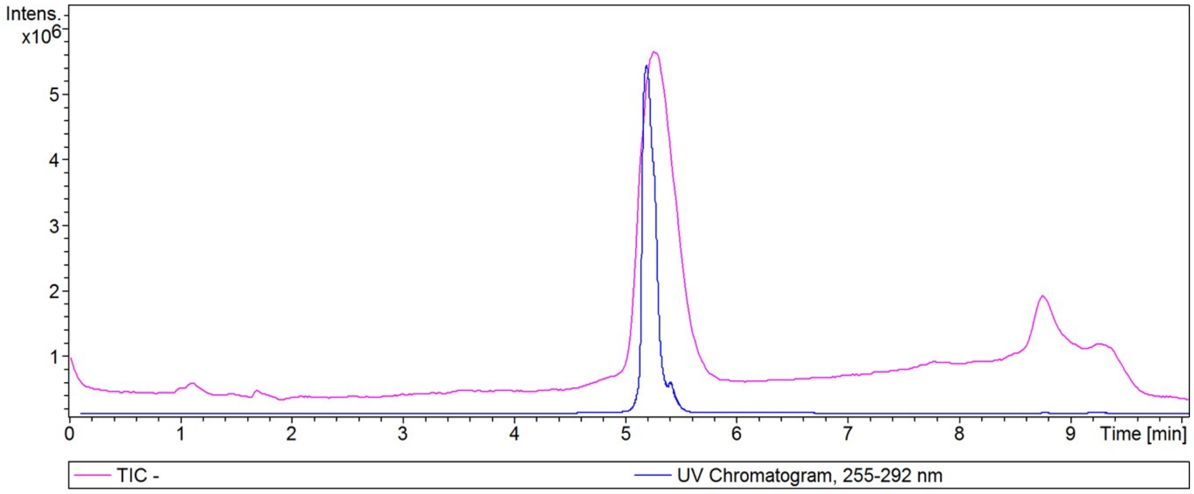

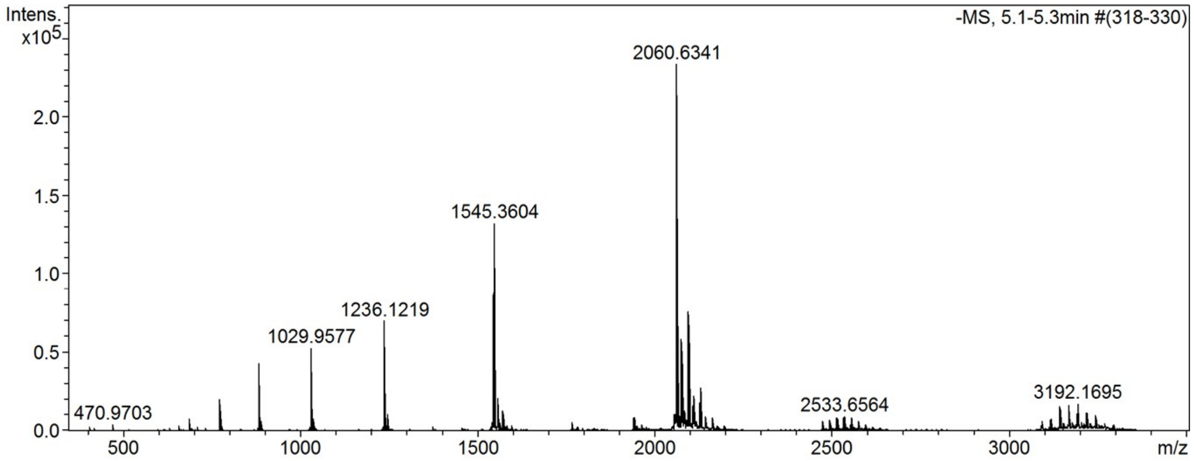

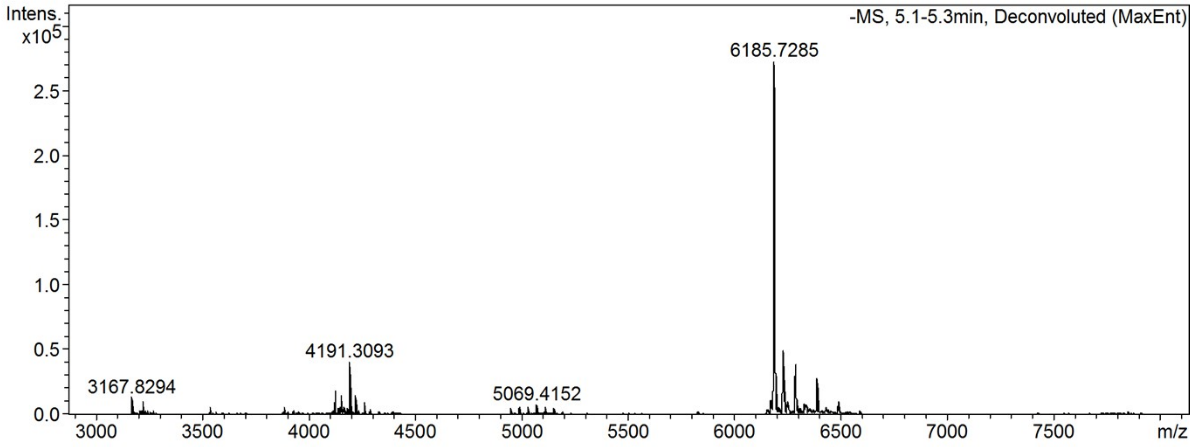

ON22: GCUAX^{PY}_{t}TACCX^{PY}_{t}TAACCCAG

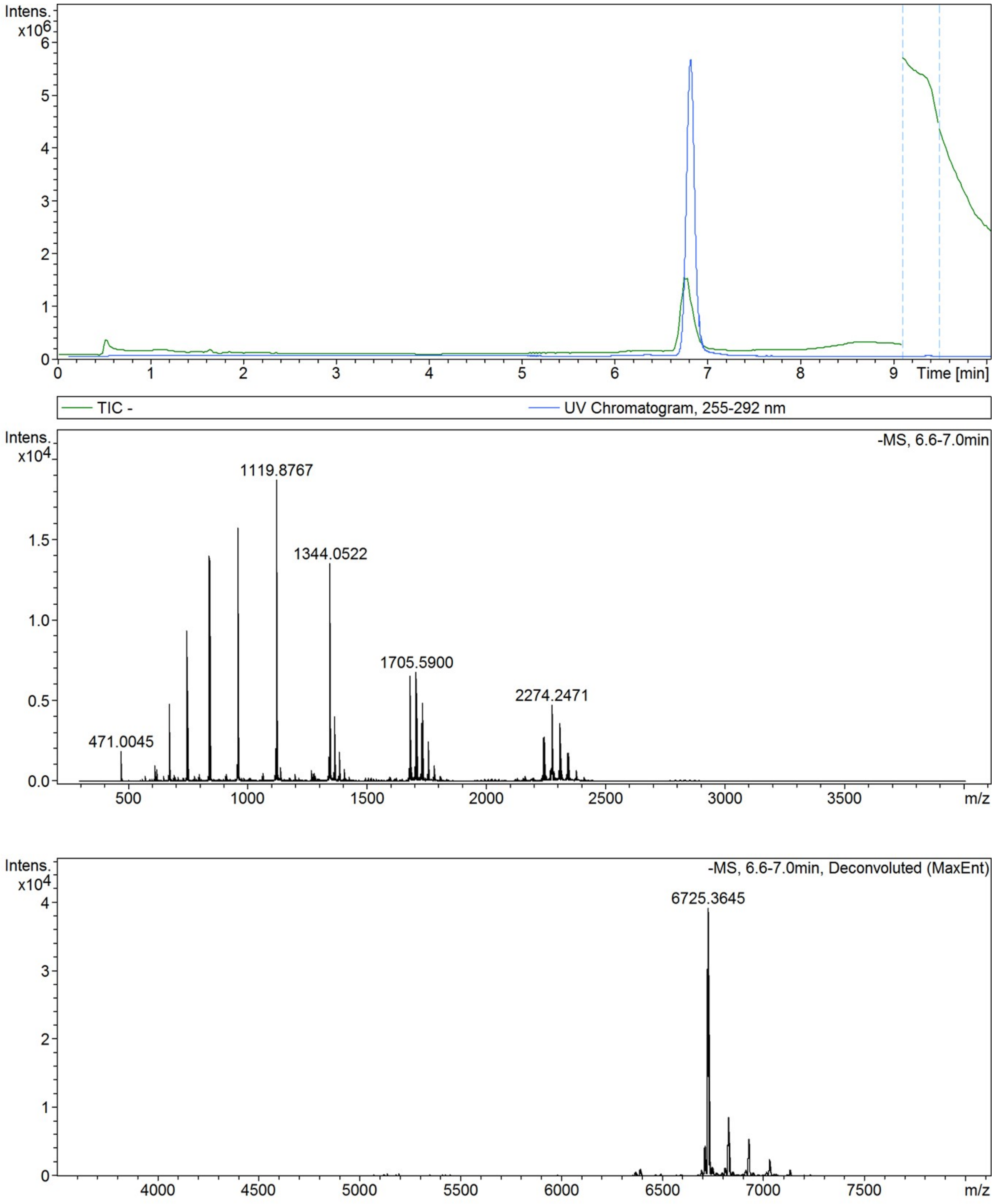

ON24: GCUAUUACCX^{PY}_{t}TAACCCAG

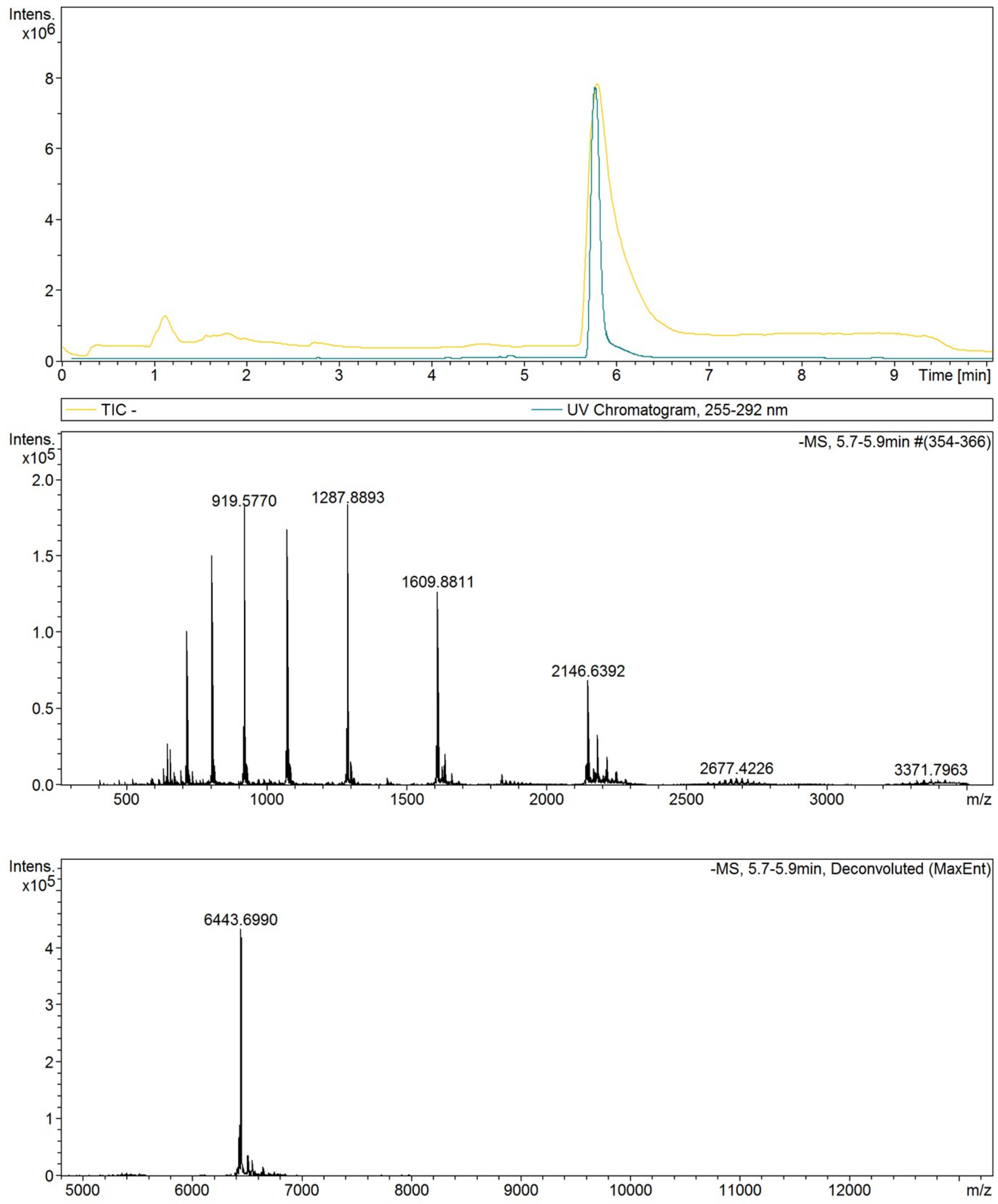

References

1. Richardson, J.A., Gerowska, M., Shelbourne, M., French, D. & Brown, T. Six-Colour HyBeacon Probes for Multiplex Genetic Analysis. *ChemBioChem* **11**, 2530-2533 (2010).
2. Bannwarth, W. Solid-Phase Synthesis of Oligodeoxynucleotides containing phosphoramidate internucleotide linkages and their specific chemical cleavage. *Helv. Chim. Acta* **71**, 1517-1527 (1988).
3. Yamamoto, I., Sekine, M. & Hata, T. One-step synthesis os 5'-azido-nucleosides. *J. Chem. Soc.-Perkin Trans. 1*, 306-310 (1980).
4. Chamberlin, K.S. Acid-aided selectivity in bromination of 1-aminoathraquinones. *Synth. Commun.* **25**, 27-31 (1995).
5. Ohrlein, R. & Baisch, G. in US patent 2009/0203931 A1 1-26 (Ciba Corporation, USA; 2009).
